# Novel Function of Cancer Stem Cell Marker ALDH1A3 in Glioblastoma: Pro-Angiogenesis through Paracrine PAI-1 and IL-8

**DOI:** 10.3390/cancers15174422

**Published:** 2023-09-04

**Authors:** Zhen Chen, Rainer Will, Su Na Kim, Maike Anna Busch, Nicole Dünker, Philipp Dammann, Ulrich Sure, Yuan Zhu

**Affiliations:** 1 Department of Neurosurgery and Spine Surgery, University Hospital Essen, University of Duisburg-Essen, 45147 Essen, Germany; 2 Center for Translational Neuro- and Behavioral Sciences (C-TNBS), University Hospital Essen, University of Duisburg-Essen, 45147 Essen, Germany; 3 Core Facility Cellular Tools, German Cancer Research Center (DKFZ), 69120 Heidelberg, Germany; 4 Institute of Anatomy II, Department of Neuroanatomy, Medical Faculty, University of Duisburg-Essen, 45147 Essen, Germany

**Keywords:** glioblastoma (GBM), endothelial angiogenesis, aldehyde dehydrogenase 1A3 (ALDH1A3), plasminogen activator inhibitor-1 (PAI-1), interleukin-8 (IL-8)

## Abstract

**Simple Summary:**

Hyper-angiogenesis is a characteristic of glioblastoma (GBM), and anti-angio-genesis is a crucial strategy to interfere with tumor progression in GBM therapy. We previously found a high expression of ALDH1A3 in proliferating vasculature in GBM patients, which was associated with poor prognosis. The present study generated two ALDH1A3-overexpressing GBM cells (oxGBMs) and demonstrated a potent pro-angiogenesis function of ALDH1A3 under the different conditions of co-culturing oxGBMs with endothelial cells in vitro and in an angiogenesis model in vivo. Moreover, we identified a mechanism underlying the oxALDH1A3-mediated pro-angiogenic effect involving the paracrine PAI-1 and IL-8 derived from oxGBMs. Blockage of PAI-1 or IL-8 hindered the hyper-angiogenesis phenotype resulting from oxALDH1A3. These findings defined a novel function of ALDH1A3 as an angiogenesis promoter in GBM, beyond its role as a well-known cancer stem cell marker, and highlighted ALDH1A3-PAI-1/IL-8 as a novel targeting signaling for future anti-angiogenesis therapy in GBM.

**Abstract:**

Hyper-angiogenesis is a typical feature of glioblastoma (GBM), the most aggressive brain tumor. We have reported the expression of aldehyde dehydrogenase 1A3 (ALDH1A3) in proliferating vasculature in GBM patients. We hypothesized that ALDH1A3 may act as an angiogenesis promoter in GBM. Two GBM cell lines were lentivirally transduced with either ALDH1A3 (ox) or an empty vector (ev). The angiogenesis phenotype was studied in indirect and direct co-culture of endothelial cells (ECs) with oxGBM cells (oxGBMs) and in an angiogenesis model in vivo. Angiogenesis array was performed in oxGBMs. RT^2^-PCR, Western blot, and double-immunofluorescence staining were performed to confirm the expression of targets identified from the array. A significantly activated angiogenesis phenotype was observed in ECs indirectly and directly co-cultured with oxGBMs and in vivo. Overexpression of ALDH1A3 (oxALDH1A3) led to a marked upregulation of PAI-1 and IL-8 mRNA and protein and a consequential increased release of both proteins. Moreover, oxALDH1A3-induced angiogenesis was abolished by the treatment of the specific inhibitors, respectively, of PAI-1 and IL-8 receptors, CXCR1/2. This study defined ALDH1A3 as a novel angiogenesis promoter. oxALDH1A3 in GBM cells stimulated EC angiogenesis via paracrine upregulation of PAI-1 and IL-8, suggesting ALDH1A3-PAI-1/IL-8 as a novel signaling for future anti-angiogenesis therapy in GBM.

## 1. Introduction

Glioblastoma (GBM) is the most devastating type of brain tumor, characterized by its aggressive and invasive growth pattern and resistance to therapy involving hyper-neoangiogenesis. Despite standard therapy incorporating surgery, radiotherapy, and chemotherapy, GBM remains incurable. The median survival time is less than two years, and the 5-year survival rate is 5.4% [1].

Hyper-angiogenesis is one of the characteristics of GBM and plays a critical role in tumor growth, invasion, and recurrence [2]. The angiogenesis process is triggered and promoted by angiogenic factors, such as vascular endothelial growth factor (VEGF), fibroblast growth factor (FGF), hypoxia-inducible factor 1-alpha (HIF1α), angiopoietin 1 (Ang-1), and angiopoietin 2 (Ang-2) that are mainly derived from tumor cells, glial cells, and stromal cells [3,4,5]. Thus, anti-angiogenesis becomes a promising strategy as an adjacent therapy besides the standard therapy for GBM [6]. In view of the current anti-angiogenic drugs such as bevacizumab (monoclonal antibodies against VEGF-A), cediranib (tyrosine kinase inhibitors against VEGFR-2), and aflibercept (decoy receptors developed from VEGFR-1), they are mostly designed to target the VEGF pathway. These anti-angiogenesis drugs slowed down the progression but did not significantly affect the poor prognosis [7,8,9,10]. GBM is a highly heterogeneous tumor. Defining a novel angiogenic signaling that targets multiple molecules in both tumor cells and ECs may improve the therapy outcome.

The aldehyde dehydrogenase (ALDH) enzyme family plays an important role in various cellular processes, including metabolism, cellular differentiation, proliferation, and the oxidative stress response [11]. Recently, ALDHs have gained much attention in cancer research due to their involvement in cancer stemness [12], resistance to therapy [13,14], and regulation of the tumor microenvironment [15]. ALDH1A3 is a member of the ALDH family and is well characterized as a cancer stem cell marker in various cancers, including lung, bile duct, prostate, colon, gastric, breast, and melanoma [16]. The impact of ALDH1A3 on GBM stem-like cells (GSCs) has been particularly noted. As a typical marker of mesenchymal (MES) GBM, the most aggressive subtype of GBM, ALDH1A3, showed the highest enzyme activity among the other ALDHs. High expression of ALDH1A3 in MES-GSCs induced mesenchymal differentiation [17], rapid intracranial tumor growth, and invasiveness [18], thereby leading to an aggressive progression of the tumor. In spite of some published data showing the implication of other members of the ALDH family in regard to angiogenesis in breast cancer cells [19] and in mesenchymal stem cells (MSCs) [20], little is known whether ALDH1A3 is involved in angiogenesis in GBM.

We have recently studied ALDH1A3 in GBM patients and found that ALDH1A3 was highly expressed in a subset of patients, which is associated with a poor prognosis. Of note, the immunoreactivity of ALDH1A3 was exclusively detected in the tumor infiltrative region, where neo-angiogenesis was active. More interestingly, we observed the expression of ALDH1A3 in the endothelial cells (ECs) of tumor vessels and glomeruloid bodies, a GBM-specific proliferating vascular structure, besides its expression in tumor cells and glial cells [21]. This expression manner found in GBM tumor tissues raised our interest to further explore whether ALDH1A3-expressing GBM tumor cells activated endothelial angiogenesis and, if so, what the underlying mechanisms were involved. To address these issues, we generated ALDH1A3-overexpressing GBM cells (oxGBMs) and investigated the endothelial angiogenesis phenotype in vitro under different conditions of co-culture of oxGBMs with ECs and in an in vivo angiogenesis model. Furthermore, the mechanism underlying oxALDH1A3-mediated pro-angiogenesis was explored.

## 2. Materials and Methods

### 2.1. Generation of ALDH1A3-Overexpressing GBM Cell Lines by Lentiviral Transduction

Two human GBM cell lines, U373 and LN229, were used to generate transduced cells overexpressing ALDH1A3. A lentiviral overexpression vector for human ALDH1A3 (ox; rwpLX305_hsALDH1A3_IRES-BsdR) was generated. The open reading frame (ORF) of human ALDH1A3 containing gateway recombination sites was synthesized in pMK (GeneArt ThermoFisher, Braunschweig, Germany). The ORF was recombined into the lentiviral expression vector rwpLX305-GW-IRES Blasti (Core Facility Cellular Tools, German Cancer Research Center, Heidelberg, Germany), under the control of a constitutive cytomegalovirus immediate early promoter (CMVie) using gateway recombination technology (ThermoFisher, Braunschweig, Germany).

Lentiviral particles were produced using a standard protocol. Briefly, HEK293FT (ThermoFisher, Braunschweig, Germany) cells were co-transfected with rwpLX305_hsALDH1A3_IRES-BsdR or empty expression vector and 2nd generation viral packaging plasmids VSV.G (plasmid #14888, Addgene, Cambridge, MA, USA) and psPAX2 (Addgene, plasmid #12260) for 2 d. Thereafter, viral supernatants were collected, filtered through a 0.45 µm filter, and used for transduction. GBM cell lines U373 and LN229 were transduced in the presence of 10 µg/mL polybrene for 24 h. After viral clearance, transduced cells were selected in Dulbecco’s Modified Eagle’s Medium (DMEM) with 10% fetal bovine serum (FBS) and 1 nM sodium pyruvate plus 5 µg/mL blasticidin (cat# A1113902; Gibco, Darmstadt, Germany). Monoclonal single-cell clones were obtained by dispensing transduced cells in single wells of a 96-well plate coated with Poly-L-Lysin (Merck, Darmstadt, Germany) using the F. sight single-cell dispensing system (Cytena, Freiburg, Germany). After the detection of single-cell growth, clones were expanded in the DMEM growth medium containing 5 µg/mL blasticidin. The transduced cells were termed ev- and oxGBMs. The expression of ALDH1A3 was validated by real-time RT2-PCR and Western blotting.

### 2.2. Real-Time RT-PCR (RT2-PCR)

Total RNA extraction, cDNA synthesis, and RT2-PCR were performed as described before [21]. Primers and corresponding annealing temperatures are listed in Table 1. The relative expression level was calculated by the 2^−ΔΔCT^ method and normalized to a reference gene, RPS13.

### 2.3. Western Blot

Total protein extraction and gel electrophoresis, blot detection, and imaging were performed as described previously [21]. Unspecific binding was blocked with a 5% non-fat milk solution and followed by incubation with primary antibodies overnight at 4 °C. The following primary antibody was used: ALDH1A3 (1:1000; cat# NBP2-15339; Novus Biologicals, Wiesbaden, Germany), PAI-1 (1:500; cat# NBP2-37532; Novus Biologicals, Wiesbaden, Germany), p-Erk1/2 (1:2000; cat# 4370; Cell Signaling Technology, Danvers, MA, USA), p-AKT (1:2000; cat# 4060; Cell Signaling Technology, Danvers, MA, USA), Erk1/2 (1:1000; cat# 9102; Cell Signaling Technology, Danvers, MA, USA), AKT (1:1000; cat# 9272; Cell Signaling Technology, Danvers, MA, USA), and GAPDH (1:2000; cat# 9664; Cell Signaling Technology, Danvers, MA, USA). For semi-quantification, the integrated optical density (IOD) of the blot bands was measured using the ImageJ software (v1.1.53t). The relative expression of a protein of interest was calculated by comparing the IOD ratio of the target protein to the reference protein GAPDH. The data were presented as a percentage of the control.

### 2.4. Angiogenesis Array

To evaluate the potential angiogenic factors released from oxGBMs, an array was carried out using a human angiogenesis array kit (cat# ARY007; R&D Systems, Wiesbaden, Germany) according to the manufacturer’s protocol. Membranes were respectively incubated with media collected from ev- and oxU373 cells after 72 h of culture. Chemiluminescent detection with multiple exposure times was performed using ImageQuant LAS 500 (GE Healthcare, Freiburg, Germany). The semi-quantification of the dots was performed using the ImageJ software (v1.1.53t). The data were presented as the mean of duplicated dots.

### 2.5. Endothelial Cell Culture

Two EC lines, human umbilical vein endothelial cells (HUVECs) and human brain microvascular endothelial cells (HBMECs), were purchased from PromoCell and Provitro, respectively. The ECs were cultured in endothelial cell growth medium with supplements (ECGM; cat# C-22010; PromoCell, Heidelberg, Germany).

### 2.6. Indirect and Direct Co-Culture of Transduced GBM Cells with Endothelial Cells and Treatment

For indirect co-culture, we first collected the media derived from the culture of transduced GBM cells. ev- or oxGBM cells (1 × 10^6^) were seeded in a dish (diameter 100 mm) and cultured in blasticidin-free DMEM growth medium for 3 d. The medium was collected, centrifuged at 2000× *g* for 10 min at 4 °C to remove cell debris, and stored at −80 °C until use. The indirect co-culture was performed by the incubation of HBMECs or HUVECs with conditioned medium (CM) containing the cell culture supernatant of transduced GBM cells (ev- and oxGBMs) and ECGM in a ratio of 1:1. Therefore, evCM and oxCM refer to the CM containing a mixture of 50% ECGM and 50% medium collected from ev- and oxGBMs, respectively.

For direct co-culture, HBMECs were pre-labeled with CellTrace™ CFSE (5 µM) (cat# C34554; Thermo Fisher Scientific, Waltham, MA, USA) according to the manufacturer’s protocol. Labeled HBMECs were then directly co-cultured with evGBMs or oxGBMs in a ratio as indicated in individual experiments in ECGM.

To determine the effects of GBM-derived PAI-1 and IL-8 on EC behavior, the following inhibitors were used: Tiplaxtinin (cat# HY-15253; MedChemExpress, Monmouth Junction, NJ, USA), a small-molecule inhibitor of PAI-1, and reparixin (cat# HY-15251; Med-ChemExpress), an allosteric inhibitor of the IL-8 receptors CXCR1 and CXCR2. Both inhibitors were dissolved in dimethyl sulfoxide (DMSO; cat# D8418; Sigma, Munich, Germany) at a 10 mM stock concentration and stored at −80 °C. In indirect co-culture, tiplax-tinin (30 µM) was added to the CM directly, and reparixin (1 µM) was pretreated with ECs for 30 min in advance and added to the CM as well. In direct co-culture, tiplaxtinin (30 µM) was pretreated with GBM cells for 6 h. Reparixin (1 µM) was pretreated with ECs 30 min before direct co-culture and added to ECGM as well in direct co-culture. Control cells received the treatment of vehicle DMSO.

### 2.7. Proliferation Assay

For indirect co-culture, EC proliferation was detected by MTT assay (cat# M6494; Invitrogen, Darmstadt, Germany) as previously described [22]. Briefly, HBMECs and HU-VECs (4000 cells/well in a 96-well plate) were incubated in evCM/oxCM without or with inhibitors or vehicle DMSO. MTT assay was performed 48 h after incubation.

For direct co-culture, CFSE-labeled HBMECs were directly co-cultured with ev- and oxGBMs (in a ratio of 1:1) in a 96-well black plate (#781668; BRAND, Wertheim, Germany) in ECGM. HBMEC proliferation was measured by detecting the fluorescence intensity of CFSE-labeled HBMEC in a plate reader (Infinite 200 Pro, Tecan, Männedorf, Switzerland) at 485 nm of excitation wavelength and 535 nm of emission wavelength. To eliminate the influence on absorbance due to the declining fluorescence intensity of CFSE labeling during the incubation, a standard curve for a linear relationship between cell number and fluorescence intensity was established. EC proliferation was quantitatively analyzed based on the standard curve.

### 2.8. Migration Assay

Scratch assay was performed to access cell migration as described before [23]. For indirect co-culture, HBMECs or HUVECs (5 × 10^5^/well) were cultured in CM with or without inhibitors. The images were acquired in six random fields of scratch per well at 0 h and 24 h after scratching. The wound healing area was calculated as the percentage of scratched area at 0 h using the ImageJ software (v1.1.53t).

For direct co-culture, cell migration was determined at 12 h after scratch of directly co-cultured GBM cells and HBMECs. The images were acquired randomly in six scratch fields per well under fluorescence microscopy (5× objective). The migrated EC with CFSE and Hoechst 33342 (cat# B2261; Sigma, Munich, Germany) was manually counted using the ImageJ software (v1.1.53t).

### 2.9. Invasion Assay

EC invasion was studied in indirect co-culture using a transwell assay as described before [23] with modifications. HBMECs and HUVECs were suspended in 200 µL of endothelial cell basal medium (ECBM) and seeded into the insert (5 × 10^4^ cells/insert in a 24-well plate) precoated with Matrigel (1 mg/mL) (cat# 356234; Corning, NY, USA). The lower chamber contained CM (700 µL/well) with or without inhibitors. After 24 h of incubation, non-invaded ECs on the up membrane of the insert were gently removed with a cotton swab. The invaded cells underneath the membrane were fixed with 4% paraformaldehyde and stained with 0.5% crystal violet. The number of invaded cells was counted on the images acquired with a 20× objective in five random fields per well.

### 2.10. Tube Formation Assay

Tube formation assay was carried out according to a previously published protocol [24], with modifications in indirect co-culture. Briefly, HBMECs and HUVECs were suspended in CM with or without inhibitors at a density of 2.5 × 10^5^ cells/mL. The cell suspension (100 µL/well in a 96-well plate) was added to the well-precoated Matrigel (50 µL), followed by incubation for 12 h and 6 h, respectively, for HUVECs and HBMECs at 37 °C. The branching points of tube-like structures were analyzed in five fields per group (5× objective) by the ImageJ software (v1.1.53t).

### 2.11. Sprouting Assay

Endothelial sprouting assay was performed in both indirect co-culture and direct co-culture using an established protocol [24] with modifications. For indirect co-culture, ECs were mixed with Cytodex 3 beads (400 cells per bead) and incubated overnight at 37 °C. After washing, the beads were resuspended in ECGM supplemented with 20% Matrigel. The mixture was quickly added to a 96-well plate and placed in the incubator for 30 min to form a gel. Thereafter, CM (100 µL/well) containing inhibitors or DMSO (0.3%) was added to the top of the gel. Sprouting was monitored and recorded at 24 h after incubation with a 10× objective. The length of 20 bead sprouts per group was measured using the ImageJ software (v1.1.53t).

For direct co-culture, CFSE-labeled HBMECs were mixed with ev- and oxGBMs in a ratio of 2:1. The mixed cells (totaling 2.4 × 10^3^) were suspended in 25 µL of ECGM containing 20% methocel solution, seeded into a U-shaped 96-well plate, and incubated overnight.

The formed spheroid was embedded individually in 50 µL of ECGM with 20% Matrigel and incubated at 37 °C for at least 30 min for gel formation. Next, ECGM (50 µL/well) was applied to the gel. EC sprouting was measured 24 h after embedding. The images of six spheroids per group were acquired using fluorescence microscopy with a 5× objective.

The length of CFSE-positive EC sprouts was measured using the ImageJ software (v1.1.53t).

### 2.12. Angiogenesis Assay on Chicken Chorioallantoic Membrane (CAM)

The angiogenesis assay model on chicken CAM was conducted as described by Palmer and Busch [25,26] with modifications. Briefly, fertilized chicken eggs were incubated in a humidified rotary incubator at 38 °C and 50% humidity for 10 d. At embryonic day 10 (ED10), the eggs were candled by shining light into the eggshell at the blunt end of the egg. The chorioallantoic vein was positioned, and a square was marked with a pencil approximately 1 cm away from the vein branching point. A hole was drilled through the blunt end of the egg into the air sac, and a window within the drawn square was opened to drop down the CAM. Through the window, 400 µL of culture medium of ev- or oxU373 cells supplemented with or without tiplaxtinin (30 µM) or reparixin (1 µM) was dropped onto the CAM. Vehicle DMSO (0.3%) was used as a control (n ≥ 10 per group). Afterward, the window was sealed, followed by incubation for 72 h. At ED13, the vasculature status of the CAM was analyzed under a stereomicroscope (Nikon SMZ 1000). The branching point of blood vessels was quantified using the ImageJ software (v1.1.53t). For histology analysis, the CM-treated CAMs were harvested and fixed in 4% paraformaldehyde, followed by paraffin embedding. The section was cut in 4 µm-thick and subjected to hematoxylin–eosin (H&E) staining. The microvessels (diameter > 10 µm) were quantified in 10 fields per CAM on microscope images (20× objective).

### 2.13. Immunohistochemistry (IHC) and Immunofluorescence (IF) Staining

IHC and double IF staining were described previously [21]. The following antibody mixtures were applied in double staining: Rabbit anti-ALDH1A3 (1:250; cat# NBP2-15339; Novus Biologicals, Wiesbaden, Germany); mouse anti-PAI-1 (1:200; cat# NBP2-37532; Novus Biologicals); mouse anti-IL-8 (1:50; cat# MAB208; R&D, Wiesbaden, Germany). Negative staining slides were incubated with nonimmune rabbit/mouse IgG in equal concentrations to the primary antibody. Counterstaining was performed with DAPI (Thermo Scientific, Schwerte, Germany). The images were acquired using an AxioImager M.2 microscope (Carl Zeiss AG, Oberkochen, Germany).

### 2.14. Database Analysis

The Pearson correlation of ALDH1A3, PAI-1, and IL-8 and with clinical prognosis presented as Kaplan–Meier curves were analyzed using the LeeY (GSE13041) dataset by Gliovis analysis (http://gliovis.bioinfo.cnio.es/, accessed on 27 August 2023) [27]. All data were accessed on 8 May 2023.

### 2.15. Statistics

Statistical analysis was performed using IBM SPSS Statistics 27 and GraphPad Prism 9. The data were presented as the mean and standard deviation (mean ± SD). Differences between two and multiple groups were analyzed by Student’s *t*-test and by one-way ANOVA followed by Tukey’s multiple comparisons test, respectively. A *p*-value less than 0.05 was considered statistically significant. Detailed statistical values of data using Prism 9 presented in each figure are reported in Supplementary Materials (Tables S1–S7).

## 3. Results

### 3.1. Overexpression of ALDH1A3 in GBM Cells Increased the Expression and Release of Pro-Angiogenic Factors

**Figure 1:**
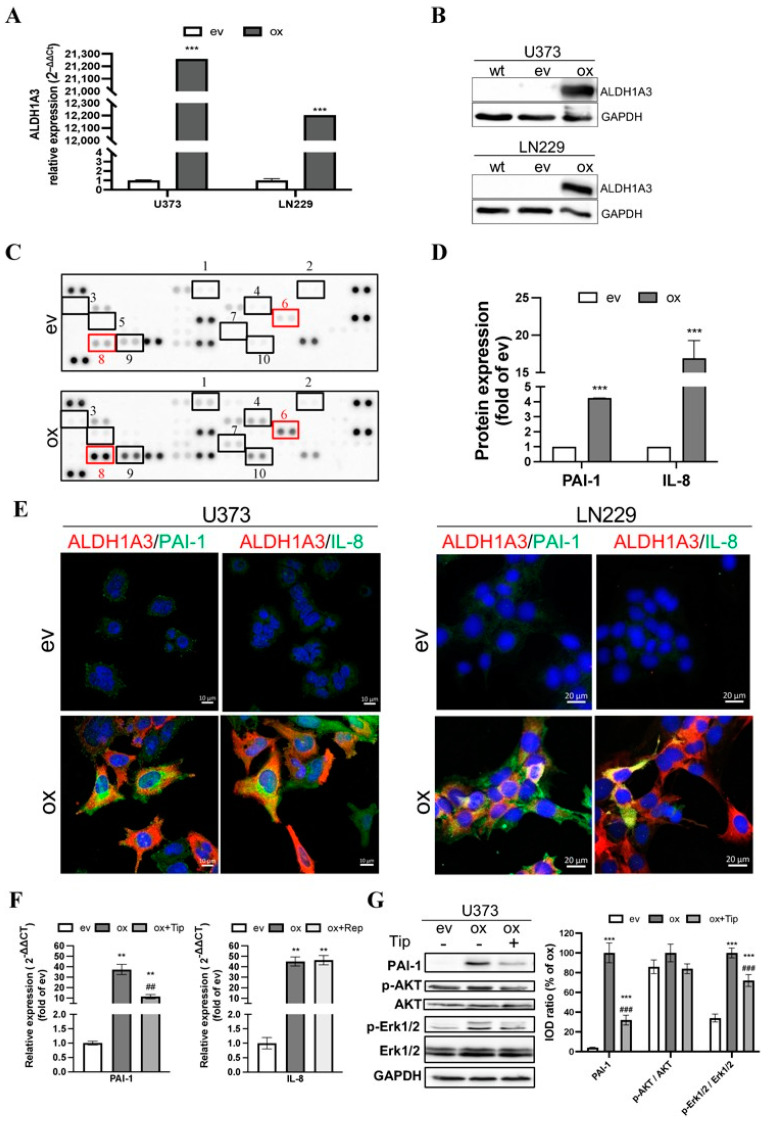
Upregulation of ALDH1A3 in oxGBM cells is associated with the increased expression and release of pro-angiogenic factors PAI-1 and IL-8. GBM cell lines were transduced with ALDH1A3 for overexpression (oxGBM) or with empty vector (evGBM). (**A**) Confirmation of up-regulation of ALDH1A3 mRNA level in oxGBM cells by RT2-PCR. (**B**) Confirmation of up-regulation of ALDH1A3 protein expression by Western blot. wt, wild-type cells. The uncropped blots are shown in Figure S7; (**C**) Angiogenesis array. The blots showed duplicated dots for 55 angiogenesis-related proteins in the media of oxU373 or evU373. 10 of 55 proteins were upregulated more than 2-fold in ox group compared to ev group (indicated by rectangle). They are: (1) Ang-1, (2) artemin, (3) TF, (4) ET-1, (5) GM-CSF, (6) IL-8, (7) PDGF-AA, (8) PAI-1, (9) PEDF, and (10) uPA. (**D**) Semi-quantification of the dots representing PAI-1 and IL-8. (**E**) Immunofluorescence staining of GBM cells. U373 (left panel) and LN229 (right panel). Co-localization of ALDH1A3 with PAI-1 and IL-8 was observed in oxGBM cells, whereas no immunoreactivity of PAI-1 and IL-8 was detected in evGBM cells. (**F**) mRNA expression of PAI-1 and IL-8 in transduced U373 cells and the effect of inhibitors. Tiplaxtinin (Tip, 30 µM) and reparixin (Rep, 1 µM) are the specific inhibitors of PAI-1 and IL-8 receptors CXCR1/2, respectively. (**G**) Detection of PAI-1 and potential signaling proteins by Western blot. IOD: optical density. The uncropped blots are shown in Figure S8. **, *p* < 0.01; ***, *p* < 0.001, compared with ev. ##, *p* < 0.01; ###, *p* < 0.001, compared with ox.

To confirm the overexpression of ALDH1A3 in transduced GBM cells, the mRNA and protein levels of ALDH1A3 were detected by RT2-PCR and Western blot, respectively. The expression of ALDH1A3 mRNA in oxU373 and oxLN229 was dramatically upregulated compared to the corresponding ev groups (Figure 1A). The overexpression of ALDH1A3 was confirmed at the protein level in oxGBMs, whereas ALDH1A3 protein expression was almost not detectable in both evU373 and evLN229 as well as in corresponding wild-type (wt) U373 and LN229 cells (Figure 1B).

To explore the potential mechanism underlying the pro-angiogenic effects induced by oxALDH1A3 in GBM cells, we performed an angiogenesis array of 55 angiogenic proteins. Figure 1C shows the image of the dots array. Semi-quantification of the blots revealed 10 of 55 upregulated proteins (≥2-fold) in the ox group compared to ev (marked in Figure 1C and Figure S1A). The upregulated proteins include (1) Ang-1, (2) artemin, (3) coagulation factor III (TF), (4) endothelin-1 (ET-1), (5) granulocyte-macrophage colony-stimulating factor (GM-CSF), (6) IL-8, (7) platelet-derived growth factor AA (PDGF-AA), (8) PAI-1, (9) pigment epithelium-derived factor (PEDF), and (10) urokinase-type plasminogen activator (uPA). These data demonstrated that overexpression of ALDH1A3 in GBM cells resulted in an elevated level of multiple angiogenic factors in the culture media. We noted that the expression of PAI-1 and IL-8 exhibited the highest abundance (Figure S1B) and a 4.3-fold and 18.6-fold upregulation of PAI-1 and IL-8, respectively, in the ox group (Figure 1D, *p* < 0.001 for both PAI-1 and IL-8). The database study using a publicly available microarray dataset (GSE13041) [28] supported a significant correlation of the transcriptional level of PAI-1 (encoded by the gene *serpin family E member 1*, *SERPINE1*) and IL-8 (encoded by the gene *C-X-C motif chemokine ligand 8*, *CXCL8*) with that of ALDH1A3 (Figure S2A). The higher expression of PAI-1 and IL-8 in GBM patients also predicted poor prognosis (HR = 0.75, 0.61, and 0.84 and *p* = 0.1831, 0.0246, and 0.4235, respectively, for ALDH1A3, PAI-1, and IL-8) (Figure S2B–D). We also explored the correlation between ALDH1A3 and other upregulated factors found in the angiogenesis array and the survival correlation in the same dataset (Figure S3). Among them, only PEDF showed a significant correlation with ALDH1A3 but without a significant difference in the survival curve with the optimal cutoff for high vs. low expression. Next, we checked the association of cellular expression of ALDH1A3 with PAI-1 or with IL-8 in cultured GBM cells. Immunofluorescence staining revealed co-expression (merged images) of ALDH1A3 (red) with PAI-1 (green) or with IL-8 (green) in oxU373 and oxLN229 cells, whereas no immunoreactivity of ALDH1A3 and PAI-1 and IL-8 was detected in evU373 and evLN229 cells (Figure 1E), which confirmed the findings shown by Western blot (Figure 1B). To confirm and verify the specificity of the upregulation of PAI-I and IL-8 resulting from oxALDH1A3, the expression of PAI-I and IL-8 was detected in the presence of the specific inhibitors. Both mRNA (Figure 1F) and protein (Figure 1G) levels of PAI-I were significantly upregulated in oxU373 cells compared with evU373 cells (*p* < 0.001 for both mRNA and protein), which was concomitantly accompanied by an increase in protein expression of p-Erk1/2 but not p-Akt. Treatment of oxU373 with a specific PAI-1 inhibitor, tiplaxtinin, significantly reduced the expression of PAI-1 at both mRNA (Figure 1F) and protein levels, as well as the protein level of p-Erk1/2 (Figure 1G).

The expression of total AKT and Erk1/2 did not differ among the tested groups. Moreover, IL-8 was also significantly upregulated in oxU373 cells, which was not affected by the treatment with reparixin (Figure 1F). Given that reparixin is an allosteric inhibitor of IL-8 receptors CXCR1/2, this is not surprising and indeed suggests that IL-8 derived from ox-GBMs may act in a paracrine manner, but not autocrine, on neighboring cells that express enriched IL-8 receptors.

### 3.2. Indirect Co-Culture Stimulated Endothelial Angiogenesis Involving Paracrine PAI-1 and IL-8

**Figure 2:**
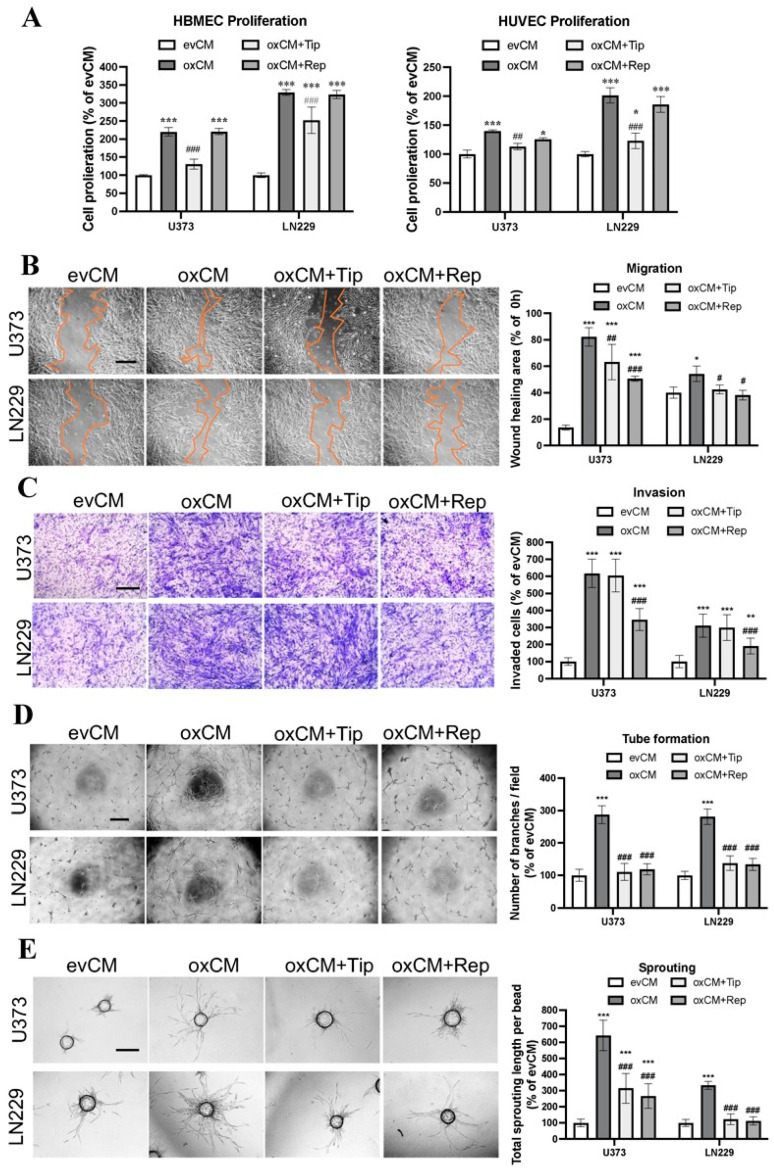
Overexpression of ALDH1A3 in GBM cells activated endothelial angiogenesis in indirect co-culture with endothelial cells (ECs), which was reversed by treatment with respective inhibitors of PAI-1 or IL-8 receptors CXCR1/2. Indirect co-culture was performed by culture of HBMECs in a conditioned medium (CM) containing the media derived from evGBMs or oxGBMs and ECGM in a ratio of 1:1. PAI-1 inhibitor tiplaxtinin (Tip, 30 µM) and CXCR1/2 inhibitor reparixin (Rep, 1 µM) or vehicle DMSO (0.1%) was added to CM, followed by EC behavior study. All data were reproduced in three independent experiments. (**A**) Proliferation assay in HBMEC and HUVEC. Indirect co-culture of HBMECs and HUVECs with CM derived from oxU373 and oxLN229 stimulated EC proliferation, which was completely reversed by the treatment of tiplaxtinin, not by reparixin. (**B**) Scratch assay in HBMEC. Left panel: images were acquired 24 h after scratching. Scale bar: 200 µm. Right panel: quantitative analysis. Culture of HBMECs with CM derived from oxU373 or oxLN229 (oxCM) significantly promoted HBMEC migration, which was reversed by the treatment of tiplaxtinin and reparixin, respectively. (**C**) Transwell invasion assay in HBMEC. Left panel: Representative images of invaded cells were acquired after 24 h of incubation. Scale bar: 100 µm. Right panel: quantitative analysis. Culture of HBMECs with oxCM accelerated HBMEC invasion. This effect was significantly inhibited by the treatment of reparixin but not by tiplaxtinin. (**D**) Tube formation assay in HBMEC. Left panel: representative images of tube formation. Scale bar: 200 µm. Right panel: quantitative analysis of branching points per field. Tube formation in HBMECs was stimulated by incubation with oxCM, which was completely diminished by both inhibitors. (**E**) Sprouting assay in HBMEC. Left panel: representative images of sprouting in HBMECs after 24 h of co-culture. Scale bar: 100 µm. A pronounced increase in sprouting was observed in HBMECs cultured in oxCM. Tiplaxtinin and reparixin suppressed the sprouting effect resulting from oxCM. *, *p* < 0.05; **, *p* < 0.01 and ***, *p* < 0.001, compared with evCM. #, *p* < 0.05; ##, *p* < 0.01 and ###, *p* < 0.001, compared with oxCM.

**Figure 3:**
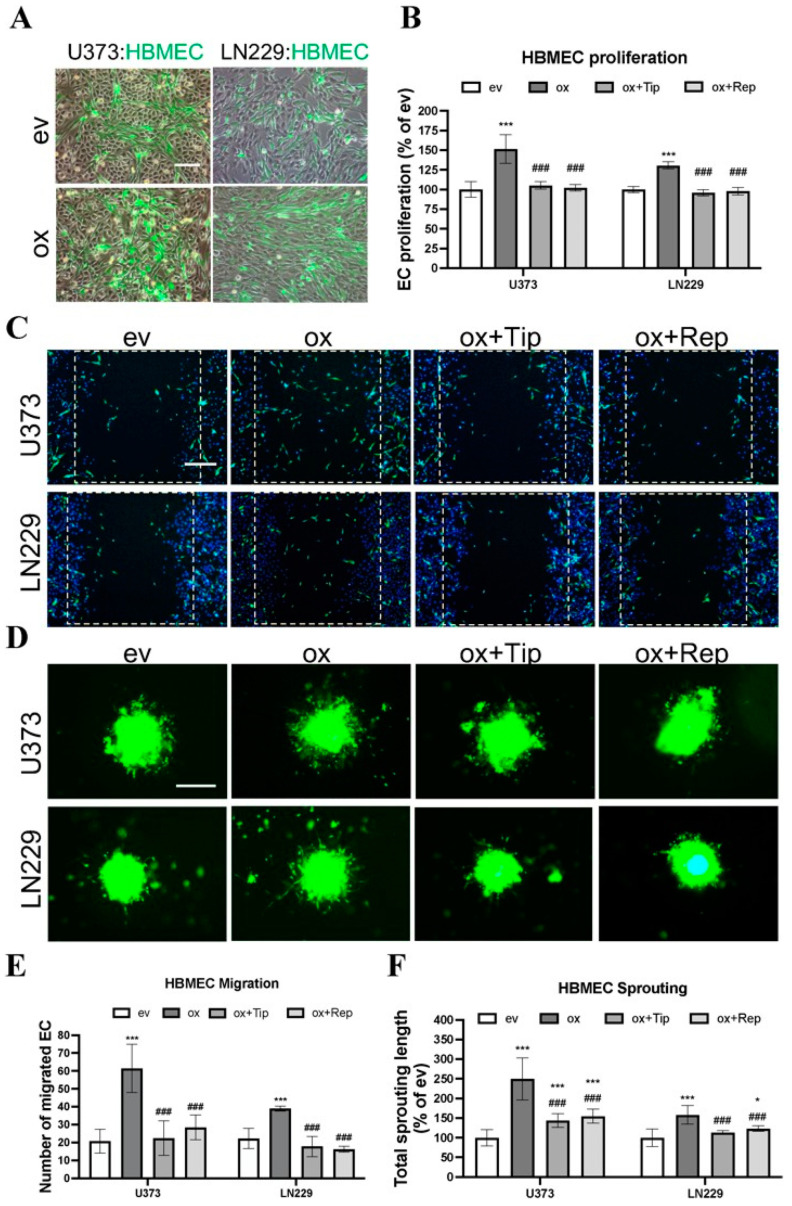
Overexpression of ALDH1A3 in GBM cells stimulated EC proliferation, migration, and sprouting in direct co-culture with endothelial cells (ECs), which was inhibited by treatment with the specific inhibitors of PAI-1 or IL-8 receptors CXCR1/2. Direct co-culture was performed by co- culture of CellTrace™ CFSE pre-labeled HBMECs with transduced U373 and LN229 in a ratio of 1:1 in ECGM. To inhibit PAI-1, tiplaxtinin (Tip, 30 µM) was pretreated with GBM cells 6 h in advance. For IL-8 receptor inhibition, reparixin (Rep, 1 µM) was pretreated with ECs 30 min in advance and added to ECGM in the co-culture, as well. All data were reproduced in three independent experiments. (**A**) Representative images of directly co-cultured CellTrace™ CFSE-labeled HBMEC (green) with transduced U373 and LN229. Scale bar: 100 µm. (**B**) HBMEC proliferation. Direct co-culture of oxGBMs with HBMECs promoted the proliferation of HBMEC, which was completely reversed by the treatment of tiplaxtinin and reparixin. The fluorescence intensity of CFSE-labeled HBMECs, reflecting cell proliferation, was detected 48 h after direct co-culture at 485 nm of excitation wavelength and 535 nm of emission wavelength. (**C**) HBMEC migration. The images were acquired after 12 h of direct co-culture. Cell nuclei were stained with Hoechst 33342 to show both GBMs and HBMECs, while CellTrace™ CFSE-labeled HBMECs displayed green fluorescence. Scale bar: 200 µm. (**D**) HBMEC sprouting. CFSE-labeled HBMECs were directly co-cultured with GBM cells in a ratio of 2:1 with or without inhibitors. The images were acquired after 24 h of direct co-culture. The sprouts with green fluorescence are exclusively from CellTrace™ CFSE-labeled HBMECs. Scale bar: 200 µm. (**E**) Quantitative analysis of migrated HBMEC. The migrated green fluorescence-labeled HBMECs were counted in 6 fields per group. Direct co-culture of oxGBMs with HBMECs markedly accelerated HBMEC migration. This effect was almost entirely inhibited in the presence of tiplaxtinin and reparixin. (**F**) Quantitative analysis of the length of sprouts in HBMEC. The length of sprouts was measured for 20 sprouts per group using the ImageJ software (v1.1.53t). *, *p* < 0.05 and ***, *p* < 0.001, compared with ev. ###, *p* < 0.001, compared with ox.

The indirect co-culture was carried out by culturing ECs in CM derived from evGBMs or oxGBMs. The key angiogenesis phenotypes of ECs, including proliferation, migration, invasion, tube formation, and sprouting, were evaluated. Proliferation assay revealed a 220% (*p* < 0.001) and 329% (*p* < 0.001) increase in the proliferation of HBMECs, respectively, after incubation with CM derived from oxU373 and oxLN229 (oxCM) compared with the corresponding evCM groups (Figure 2A, left panel). A significantly increased proliferation was also observed in HUVECs (Figure 2A, right panel). Moreover, a 6.0-fold (*p* < 0.001) and 1.4-fold (*p* < 0.05) increase in migration were detected in HBMECs cultured with oxCM derived from oxU373 and oxLN229, respectively (Figure 2B). Invasion assay revealed 6.2-fold (*p* < 0.001) and 3.1-fold (*p* < 0.001) more invaded HMBEC in oxCM from respective oxU373 and oxLN229 (Figure 2C). Incubation of HBMECs with oxCM also largely promoted tube formation, as evidenced by a nearly 300% increase in tube branching points (*p* < 0.001) (Figure 2D) as well as sprouting (Figure 3E). These indirect co-culture data suggested that overexpression of ALDH1A3 induced a pro-angiogenesis effect in ECs via the secreted soluble factors.

To evaluate whether PAI-1 and IL-8 are involved in this effect as key angiogenic factors, the specific PAI-1 inhibitor tiplaxtinin and the IL-8 receptor CXCR1/2 inhibitor reparixin were added to oxCM. We found that tiplaxtinin and reparixin commonly or differently affected different endothelial behaviors. Tiplaxtinin (Figure 2A) but not reparixin reversed the proliferation effect of oxCM, whereas reparixin but not tiplaxtinin suppressed the increase in invasion in oxCM-incubated HBMECs (Figure 2C). Interestingly, both inhibitors partially reversed the migration effect (Figure 2B), but completely abolished tube formation (Figure 2D) and sprouting (Figure 2E) mediated by oxCM. The pro-angiogenic effect of oxCM and the rescue effect of inhibitors were similarly observed in the indirect co-culture of HUVECs with oxCM (Figure S4). Interestingly, the combinational treatment of both inhibitors was more effective in suppressing the proliferation and migration of HUVEC cultured in oxCM (Figure S5). Additionally, we also assessed the impact of these inhibitors on the evCM-treated group and observed no discernible effects on EC behaviors (Figure S6). These findings indicate PAI-1 and IL-8 as key angiogenic factors involved in the pro-angiogenesis mediated by oxGBMs.

### 3.3. Direct Co-Culture of oxGBMs with ECs Produced Pro-Angiogenic Effect on HBMEC, Which Was Reversed by the Treatment of PAI-1 and IL-8 Receptors Inhibitors

Next, we studied the EC angiogenic phenotype in direct co-culture, which allows direct interaction of GBM cells with ECs, mimicking an in vivo tumor environment. To distinguish HBMECs from co-cultured GBM cells, CFSE, a long-retaining green fluorescence tracker for living cells, was used to label HBMECs prior to direct co-culture. Figure 3A shows CFSE-labeled HBMECs (green) directly co-cultured with transduced U373 or with LN229 cells in a ratio of 1:1. The angiogenic phenotype of HBMEC, including proliferation, migration, and sprouting, was investigated under this established direct co-culture condition. As presented in Figure 3B, HBMECs grew more rapidly when directly co-cultured with oxU373 and oxLN229 cells than with corresponding evGBMs (151.5% and 130.6%, respectively, for U373 and LN229; *p* < 0.001). Of note, this proliferative effect was completely reversed by the treatment with tiplaxtinin and reparixin (*p* < 0.001). The images acquired 12 h after scratching revealed more migrated HBMECs (green) when directly co-cultured with oxU373 and oxLN229 (Figure 3C). A remarkable increase in HBMEC sprouting appeared after 24 h of direct co-culture with oxU373 and oxLN229 (Figure 3D). Quantitative analysis of the migration images demonstrated a 6-fold (*p* < 0.001) and 4-fold (*p* < 0.001) increase in migrated HBMECs when co-cultured with oxU373 and oxLN229, respectively, which was entirely suppressed by the treatment of both inhibitors (Figure 3E). Analysis of the length of EC sprouts indicated 2.5-fold (*p* < 0.001) and 1.5-fold (*p* < 0.001) longer sprouts in HBMECs co-cultured with oxU373 or with oxLN229, respectively, and this effect was restored to the basal level in the corresponding evGBM groups in the presence of the respective inhibitors tiplaxtinin and reparixin (Figure 3F). These findings are consistence with that observed in indirect co-culture, indicating a potent pro-angiogenic role of ALDH1A3 via PAI-1 and IL-8.

### 3.4. oxGBMs-Derived Culture Media Stimulated Endothelial Angiogenesis In Vivo, Which Was Suppressed by Treatment with Inhibitors of PAI-1 and IL-8 Receptors

**Figure 4:**
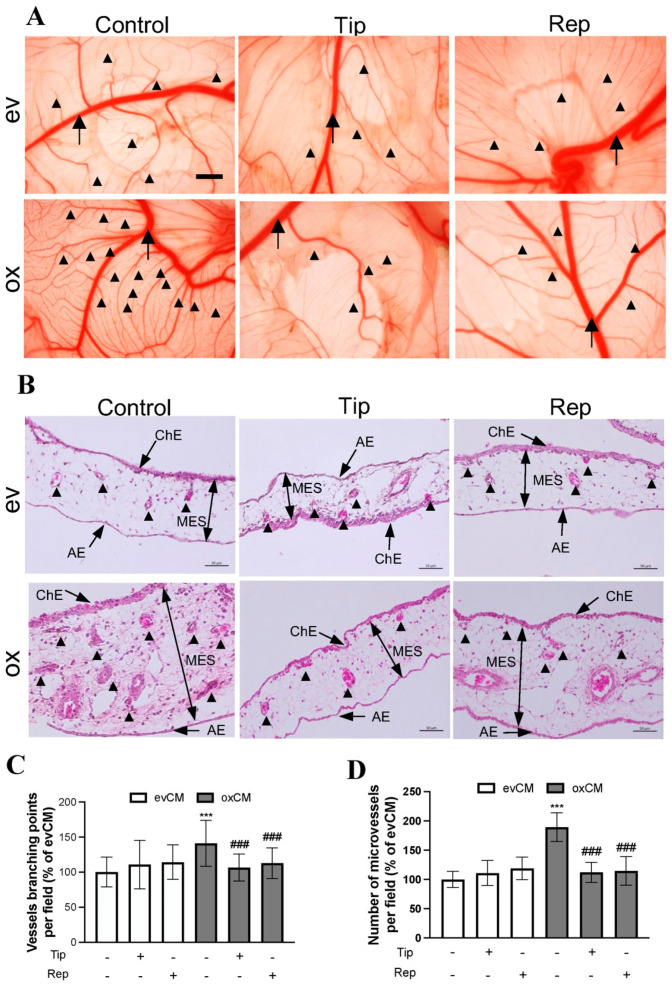
oxGBM-derived culture media stimulated angiogenesis on CAM, which was fully rescued by the treatment of tiplaxtinin and reparixin. CAM was incubated with the culture media derived from evU373 or oxU373 cells with or without tiplaxtinin (Tip, 30 µM) or reparixin (Rep, 1 µM) or DMSO (as a vehicle control, 0.1%) for 72 h. (**A**) Microscopy view of the vasculature structure on CAM. More enriched microvessel network was clearly visible in the ox group, which was significantly reduced in tiplaxtinin- and reparixin-treated CAMs. The images were acquired using a stereo microscope on ED13 (scale bar: 1 mm). Stem vessel (arrow); branched microvessel network (arrow heads). (**B**) Histological features of CAM after hematoxylin-eosin (H&E) staining. The CAM consists of the chorionic epithelium layer (ChE), allantoic epithelium (AE) layer, and the mesenchymal (MES) layer (arrows). Microvessel (arrowheads) density was much higher in the MES layer of ox section compared to ev section, which was clearly reduced in tiplaxtinin- and reparixin-treated sections. Scale bar: 50 µm. (**C**) Quantitative analysis of branching point of vessels based on microscopy images. The number of branching points and microvessels was counted by the ImageJ software (v1.1.53t) in 3 fields/CAM (n = 10 CAM/group). (**D**) Quantitative analysis of microvessel numbers based on H&E-stained CAM sections. Microvessel number was counted manually on H&E-stained CAMs. 10 fields/section (n = 6 sections/group). ***, *p* < 0.001, compared with ev. ###, *p* < 0.001, compared with ox.

To validate our in vitro findings, we employed an in vivo angiogenesis model in chicken CAM. Figure 4A shows representative images of the vasculature on CAM 3 d after treatment with media derived from transduced U373 cells. Along with the stem (big) vessel (arrow), a denser and higher-branched microvessel network (arrowheads) was visualized in the ox group compared to that in the ev group. This hyper-angiogenic phenotype was attenuated in the presence of the inhibitors tiplaxtinin or reparixin. Further histological studies were performed on CAM sections. H&E staining revealed the histological and vascular features of the CAM (Figure 4B). We observed a higher microvessel density in the ox group than that in the ev group, which was reduced in the presence of individual inhibitors in the treatment media. Interestingly, a much thicker mesenchymal (MES) layer was observed on the section from ox media-incubated CAM. This may be a result of the proliferation of mesenchymal cells and microvessels. Figure 4C and Figure 4D, respectively, show the quantitative analysis of the branching points (based on CAM images in Figure 4A) and the number of microvessels (based on histological sections in Figure 4B). 150% of the increase in vessel branching point (Figure 4C, *p* < 0.001) and 189% of the higher microvessel density (Figure 4D, *p* < 0.001) were found in the ox group compared with the corresponding ev group. This increase in both parameters of angiogenesis was completely diminished by the treatment of the individual inhibitors of PAI-1 and IL-8 receptors (both *p* < 0.001), whereas the basal level of vascular branching point and number of microvessels in the corresponding ev group was not affected by inhibitors (Figure 4C,D). These in vivo findings strongly supported the data from our in vitro study.

### 3.5. Co-Expression of ALDH1A3 with PAI-1 or with IL-8 in Tumor Vessels and Tumor Cells of GBM

**Figure 5:**
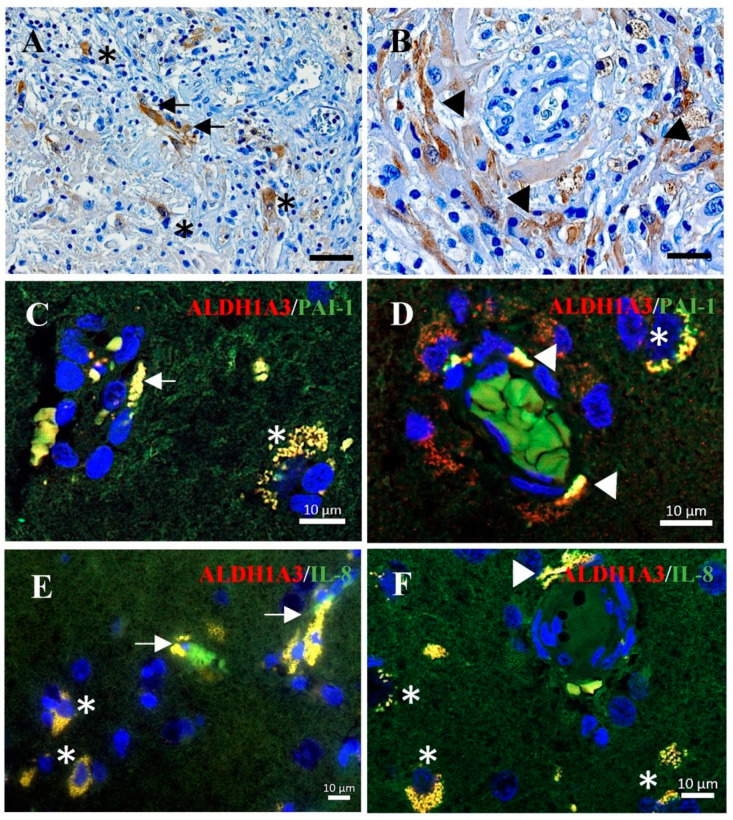
Immunohistochemistry and immunofluorescence staining on GBM sections. (**A**,**B**) Immunohistochemistry staining of ALDH1A3. The immunoreactivity of ALDH1A3 was detected in vessels (arrows in (**A**)) and peripheral cells of glomeruloid (arrowheads in (**B**)) as well as in tumor cells (asterisks in (**A**)). Scale bar: 50 µm in (**A**) and 20 µm in (**B**). (**C**–**E**) Double-immunofluorescence staining of ALDH1A3 (red) with PAI-1 (green) (**C**,**D**) or with IL-8 (green) (**E**,**F**). The co-localization of ALDH1A3 with PAI-1 or with IL-8 immunoreactivity was detected in microvessels (arrows), glomeruloid (arrowheads), and tumor cells (asterisks) in GBM sections.

Immunohistochemistry staining on GBM sections confirmed the expression of ALDH1A3 in the endothelial cells (arrows, Figure 5A) and in the peripheral cells of proliferating glomeruloid (arrowheads in Figure 5B), as well as in some tumor cells (asterisks in Figure 5A). This cellular expression pattern of ALDH1A3 is in agreement with our previous findings [21]. Double immunofluorescence staining demonstrated a co-localization of the immunoreactivity of ALDH1A3 (red) with PAI-1 (green) (Figure 5C,D) or with IL-8 (green) (Figure 5E,F) in vessels (arrows), the peripheral cells of glomeruloid bodies (arrowheads), and in tumor cells (asterisks).

## 4. Discussion

ALDH1A3 is a well-known cancer stem cell marker in various cancers [16]. In GBM, ALDH1A3 is a key driver in the transition of proneural to mesenchymal GBM, and the latter is the most aggressive subtype of GBM [17]. Thus, ALDH1A3 is also used as a prognostic marker for GBM [18,29]. We have recently reported a significant upregulation of ALDH1A3 in a subset of GBM patients, which was associated with a poor prognosis. Of note, ALDH1A3 was dominantly detected in the endothelial cells of tumor vessels and in glomeruloid, a proliferative vasculature typically seen in GBM, in the infiltration zone where an active neo-angiogenesis appeared [21]. However, so far, little is known about the functional role of ALDH1A3 in tumor angiogenesis beyond its role as a stem cell marker. We hypothesized that ALDH1A3 may play a crucial role in neo-angiogenesis in GBM. In the present study, we provided evidence, for the first time to our knowledge, for the potent pro-angiogenesis function of ALDH1A3 by using both indirect co-culture and direct co-culture of ALDH1A3-overexpressing GBM cells with ECs in vitro and by applying an angiogenesis model in vivo. Moreover, we investigated the underlying mechanism involved in the pro-angiogenesis effect of ALDH1A3. Overexpression of ALDH1A3 in GBM cells resulted in a significant upregulation of PAI-1 and IL-8 mRNA and protein expression in oxGBMs and a consequential increase in the release of PAI-1 and IL-8 that in turn acted on ECs in a paracrine manner, thereby stimulating neo-angiogenesis. Blockage of PAI-1 and IL-8 receptors CXCR1/2 by specific and respective inhibitors rescued the activated angiogenesis phenotype both in vitro and in vivo. Our findings defined ALDH1A3 as a novel angiogenesis promoter, which may highlight this molecule as a potential target for anti-angiogenesis strategies in GBM therapy in the future.

It is well known that the interaction of tumor cells (TCs) with neighboring cells has a significant impact on tumor progression. This interaction occurs in cell–cell direct contact and/or in a paracrine manner. The present study used indirect and direct co-culture of oxGBMs with ECs, which mimicked the tumor microenvironment for the interaction between TCs and ECs. The indirect co-culture model simulated the TC–EC interaction in a paracrine manner, whereas direct co-culture allowed TC–EC contact directly and simultaneously. Under both co-cultured conditions, we observed a significantly activated angiogenesis phenotype, including increased EC proliferation, migration, invasion, tube formation, and sprouting, in ECs treated with oxCM or directly co-cultured with oxGBMs. The oxGBM-mediated angiogenesis was confirmed in an angiogenesis model, as evidenced by denser and more highly branched microvessels in ox media-treated CAM. These data are supportive of the notion that TCs can trigger ECs to stimulate EC angiogenesis [30,31,32] and also indicate that ALDH1A3 is not only a stem cell marker but also a novel angiogenesis promoter in GBM.

Hyper-angiogenesis is a typical feature of GBM, which is driven by aberrant expression of angiogenic factors in the tumor microenvironment. In MES-GBM cells, the levels of VEGF, Ang-1, and Ang-2 were significantly elevated compared with PN-GBM cells, which was associated with a high expression of ALDH1A3 [33]. In the present study, we detected an upregulation of multiple angiogenic factors by an angiogenesis array in the media derived from oxGBMs culture. Many of them (e.g., Ang-1, ET-1, GM-CSF, PDGF-AA, IL-8, PAI-1, and uPA) are well-known factors implicated in tumor angiogenesis. Considering the expression abundance and the upregulation extent shown in the array, we paid particular attention to PAI-1 and IL-8. PAI-1 is a serine protease inhibitor and has been shown to stimulate angiogenesis by promoting migration, survival, and proliferation of ECs via inhibiting plasminogen activation and protecting ECs from FasL-dependent extrinsic apoptosis [34]. Overexpression of PAI-1 is associated with poor prognosis in various cancer types, including GBM [35,36]. IL-8, also known as CXCL8, is a pro-inflammatory chemokine in the CXC family. Previous studies have demonstrated its role in promoting angiogenesis and invasion in GBM [37,38,39,40,41]. Moreover, the level of IL-8 in the serum is correlated with tumor progression [42,43]. Our database study also provided supportive evidence for the association of ALDH1A3 and PAI-1/IL-8 and their pivotal role in the prognosis of GBM (Figure S2). In line with these published data and our own findings, we assumed that PAI-1 and IL-8 are the downstream molecules targeted by ALDH1A3, and both are important intermediates of pro-angiogenesis function mediated by oxALDH1A3. RT2-PCR, and Western blot further confirmed the increase in mRNA and protein expression of PAI-1 and IL-8 in oxGBMs. Intriguingly, the upregulation of PAI-1 in oxU373 cells was significantly reversed by the treatment of a specific PAI-1 inhibitor, tiplaxtinin, at both mRNA and protein levels. The data suggest that mRNA/protein expression of PAI-1 might be autonomously regulated, which is interfered with by tiplaxtinin. Tiplaxtinin has been considered a functional inhibitor of PAI-1. The inhibitory effect of tiplaxtinin is thought to be mediated by its specific binding to the active conformation of PAI-1 at the vitronectin binding site (in the central β-sheet A cleft), thereby reversibly inactivating PAI-1 [44,45]. Most studies reported the functional inhibitory effect of tiplaxtinin on secreted PAI-1. However, it is noteworthy that tiplaxtinin may target not only secreted PAI-1 but also intracellular PAI-1. It is speculated that tiplaxtinin itself may regulate the expression of PAI-1. It is certainly of interest in the future to further explore the role of tiplaxtinin in regulating intracellular PAI-1 in oxGBM. Of note, treatment with the IL-8 receptor inhibitor reparixin did not affect the expression of IL-8 mRNA in oxU373 (Figure 1F). This is not surprising but rather suggests that IL-8 expression is regulated by an IL-8 autocrine-independent mechanism because reparixin is a selective inhibitor that blocks the IL-8 receptors. It is known that enriched IL-8 receptor-expressing cells include ECs but not GBM cells [46,47,48]. Thus, reparixin could markedly suppress oxALDH1A3-mediated angiogenesis in ECs without affecting the cell-autonomous expression of IL-8 in oxGBMs. These data demonstrated that PAI-1 and IL-8 were truly downstream targets of ALDH1A3.

Next, we investigated the role of PAI-1 and IL-8 in oxGBM-driven EC angiogenesis after the treatment of specific inhibitors. In indirect co-culture, increased EC migration, invasion, tube formation, and sporting resulting from oxCM treatment were partially but significantly reversed by both inhibitors, whereas the EC proliferative effect of oxCM was attenuated only by tiplaxtinin but not by reparixin, suggesting a common but also different role of PAI-1 and IL-8 on oxALDH1A3-driven EC behaviors (Figure 2). Interestingly, combinational treatment of both inhibitors entirely inhibited the proliferation and migration effects to their basal levels, as shown in evCM, indicating a synergistic inhibitory effect derived from the combinational treatment (Figure S5). In direct co-culture, oxGBM-mediated increases in EC proliferation, migration, and sprouting were completely abolished in the presence of both inhibitors in direct co-culture (Figure 3). Thus, the blockage effect of inhibitors on angiogenesis seems to be more obviously observed in the direct co-culture model than in the indirect co-culture model. It is assumed that the inhibitors may exhibit a more powerful inhibitory effect by interfering with both autocrine and paracrine effects of PAI-1 and IL-8 in direct co-culture, whereas only paracrine angiogenic factors could be affected by the inhibitors in indirect co-culture. Despite the firmly observed inhibitory effects of specific inhibitors on angiogenesis resulting from oxALDH1A3, we could not rule out the possibility of off-target effects of the inhibitors. Application of gene editing approaches (e.g., siRNA, shRNA, or CRISPRi) may provide further evidence and avoid any potential off-target effects of small molecules. However, the gene-editing approach may not be favored for silencing PAI-1 in the present study due to its dual functions in angiogenesis.

It is known that PAI-1 plays a pro-angiogenic role through binding to uPA [49], whereas binding of PAI-1 to vitronectin shows an anti-angiogenic function [50]. Globally silencing PAI-1 may affect the binding of PAI-1 to both uPA and vitronectin. Of note, tiplaxtinin specifically blocks the binding of PAI-1 to uPA without affecting the binding of PAI-1 to vitronectin [44], suggesting that tiplaxtinin is a feasible small molecule to specifically inhibit PAI-1-induced angiogenesis.

**Figure 6:**
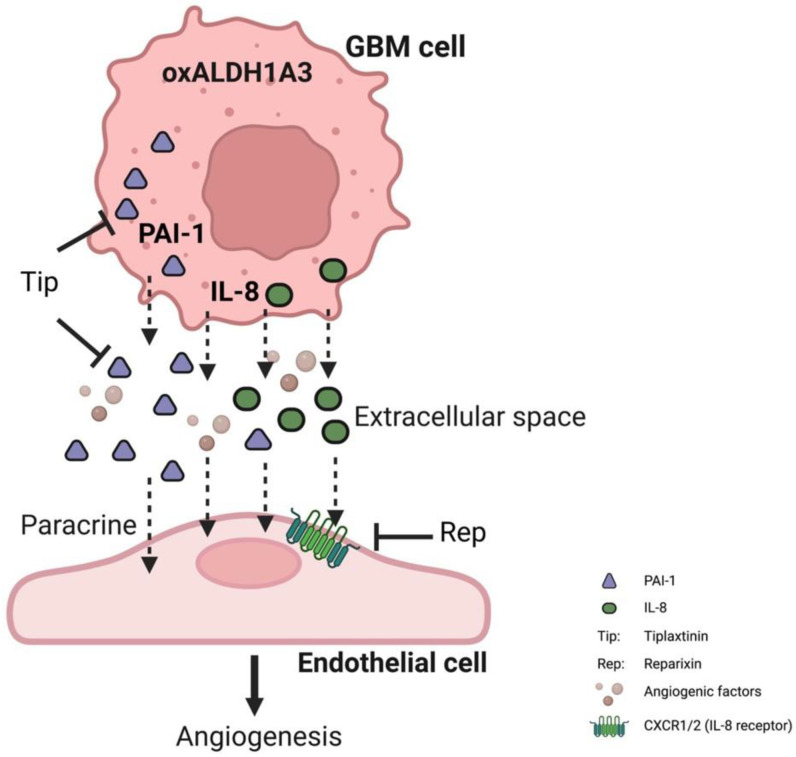
Schematic illustration of the pro-angiogenic function of ALDH1A3 via paracrine PAI-1 and IL-8. The overexpression of ALDH1A3 in GBM cells induces upregulation of both PAI-1 and IL-8 at the mRNA and protein levels, resulting in the subsequent release of these proteins into the extracellular compartment. Along with other angiogenic factors, the released PAI-1 and IL-8 exert their effects on ECs, triggering paracrine-mediated hyper-angiogenesis. However, the application of tiplaxtinin, a compound that promotes the cleavage of PAI-1, or reparixin, specific inhibitors of the IL-8 receptor CXCR1/2, effectively counteracted the pro-angiogenic impact caused by ALDH1A3. These findings strongly support the notion that PAI-1 and IL-8 play critical roles as downstream components of ALDH1A3 in GBM, underscoring their significance within the context of angiogenesis.

Taken together, overexpression of ALDH1A3 in GBM cells led to pro-angiogenesis in ECs via upregulation of PAI-1, IL-8, and other factors and a consequential release of these factors into extracellular space. The released angiogenic factors PAI-1 and IL-8 in turn acted on ECs leading to the activation of angiogenesis, which is blocked by the treatment of the specific inhibitors tiplaxtinin and reparixin. We believe that PAI-1 and IL-8, as the most important intermediates, together with other upregulated angiogenesis factors, synergistically contributed to the pro-angiogenesis effect derived by oxALDH1A3 in GBM (Figure 6).

## 5. Conclusions

The present study defined ALDH1A3 as a novel and potent angiogenesis promotor in GBM cells, which provided direct evidence supporting our previous findings in the GBM patient cohort study. The underlying mechanism of the pro-angiogenic effect derived from ALDH1A3-overexpressing GBM cells involves the paracrine PAI-1 and IL-8. Our findings highlight ALDH1A3-PAI-1/IL-8 as a novel targeting signaling for future anti-angiogenesis therapy in GBM.

## Figures and Tables

**Table 1:** List of primer sequences and annealing temperatures for RT2-PCR.

Primer Name	Annealing Sequence	Temperature (°C)
ALDH1A3		60
*for.*	TCTCGACAAAGCCCTGAAGT	
*rev.*	TATTCGGCCAAAGCGTATTC	
PAI-1		60
*for.*	GGTTCTGCCCAAGTTCTCCC	
*rev.*	CACCGTGCCACTCTCGTTCA	
IL-8		62
*for.*	CTTGGCAGCCTTCCTGATTT	
*rev.*	TTTCCTTGGGGTCCAGACAGA	
RPS13		60
*for.*	CGAAAGCATCTTGAGAGGAACA	
*rev.*	TCGAGCCAAACGGTGAATC	

*for.*: forward; *rev.*: reverse.

## Data Availability

The data presented in this study are available in this article (and Supplementary Material).

## Supplementary Materials

The following supporting information can be downloaded at: https://www.mdpi.com/article/10.3390/cancers15174422/s1, Table S1: ANOVA analysis table of mRNA and protein expression data presented in Figure 1; Table S2: ANOVA analysis table of indirect co-culture study data presented in Figure 2; Table S3: ANOVA analysis table of direct co-culture study data presented in Figure 3; Table S4: ANOVA analysis table of in vivo study data presented in Figure 4; Table S5: ANOVA analysis table of HUVEC data presented in Figure S4; Table S6: ANOVA analysis table of synergistic effects data presented in Figure S5; Table S7: ANOVA analysis table of effect of tiplaxtinin and reparixin on EC cultured in evCM data presented in Figure S6; Figure S1: Semi-quantification of the blots of angiogenesis array; Figure S2: The expression of ALDH1A3, PAI-1, and IL-8 is associated with poor prognosis in GBM; Figure S3: The expression and survival correlations of ALDH1A3 and angiogenesis factors in GBM; Figure S4: Indirect co-culture study of oxGBMs with HUVECs; Figure S5: Synergistic effects of tiplaxtinin and reparixin on proliferation and migration of HU-VECs in indirect co-culture model; Figure S6: Effect of tiplaxtinin and reparixin on EC cultured in evCM; Figure S7: Original immunoblots for Figure 1B; Figure S8: Original immunoblots for Figure 1G.
