# Supplementary material for: Novel Function of Cancer Stem Cell Marker ALDH1A3 in Glioblastoma: Pro-Angiogenesis through Paracrine PAI-1 and IL-8
Source: Cancers (Basel). 2023 Sep 4;15(17):4422. doi: 10.3390/cancers15174422 (PMC10487054; doi:10.3390/cancers15174422)
Supplement: Supplementary file 1 [file cancers-15-04422-s001.zip › cancers-2554568-supplementary.pdf]

Table S1. ANOVA analysis table of mRNA- and protein expression data presented in Figure 1.

ANOVA table	SS	DF	MS	F (DFn, DFd)	*p* value
**Figure 1 F_PAI-1**					
Treatment (between columns)	1404	2	702.2	F (2, 3) = 74.75	*p*=0.0028
Residual (within columns)	28.18	3	9.395		
Total	1433	5			
**Figure 1 F_IL-8**					
Treatment (between columns)	2667	2	1334	F (2, 3) = 107.9	*p*=0.0016
Residual (within columns)	37.08	3	12.36		
Total	2704	5			
**Figure 1 G_PAI-1**					
Treatment (between columns)	14624	2	7312	F (2, 6) = 175.1	*p*<0.0001
Residual (within columns)	250.5	6	41.75		
Total	14875	8			
**Figure 1 G_pAKT**					
Treatment (between columns)	456	2	228	F (2, 6) = 4.413	*p*=0.0663
Residual (within columns)	310	6	51.67		
Total	766	8			
**Figure 1 G_pErk1/2**					
Treatment (between columns)	6584	2	3292	F (2, 6) = 128.3	*p*<0.0001
Residual (within columns)	154	6	25.67		
Total	6738	8			

DF: degrees of freedom; SS: sum of squares; MS: mean square (=SS/DF); F: F distribution value; *p*: probability.

Table S2. ANOVA analysis table of indirect co-culture study data presented in Figure 2.

ANOVA table	SS	DF	MS	F (DFn, DFd)	*p* value
**Figure 2A_left panel_U373**					
Treatment (between columns)	46190	3	15397	F (3, 12) = 157.2	p<0.0001
Residual (within columns)	1175	12	97.94		
Total	47366	15			
**Figure 2A_left panel_LN229**					
Treatment (between columns)	136956	3	45652	F (3, 12) = 119.4	p<0.0001
Residual (within columns)	4589	12	382.4		
Total	141546	15			
**Figure 2A_right panel_U373**					
Treatment (between columns)	3498	3	1166	F (3, 12) = 52.88	p<0.0001
Residual (within columns)	264.6	12	22.05		
Total	3762	15			
**Figure 2A_right panel_LN229**					
Treatment (between columns)	28476	3	9492	F (3, 12) = 67.50	p<0.0001
Residual (within columns)	1688	12	140.6		
Total	30164	15			
**Figure 2B_U373**					
Treatment (between columns)	10022	3	3341	F (3, 12) = 56.77	p<0.0001
Residual (within columns)	706.2	12	58.85		
Total	10729	15			
**Figure 2B_LN229**					
Treatment (between columns)	628	3	209.3	F (3, 12) = 10.82	p=0.0010
Residual (within columns)	232.1	12	19.35		
Total	860.1	15			
**Figure 2C_U373**					
Treatment (between columns)	1812579	3	604193	F (3, 36) = 116.7	p<0.0001
Residual (within columns)	186390	36	5177		
Total	1998969	39			
**Figure 2C_LN229**					
Treatment (between columns)	296993	3	98998	F (3, 36) = 28.63	p<0.0001
Residual (within columns)	124491	36	3458		
Total	421484	39			
**Figure 2D_U373**					
Treatment (between columns)	118969	3	39656	F (3, 16) = 76.14	p<0.0001
Residual (within columns)	8333	16	520.8		
Total	127302	19			
**Figure 2D_LN229**					
Treatment (between columns)	96886	3	32295	F (3, 16) = 81.16	p<0.0001
Residual (within columns)	6367	16	397.9		
Total	103253	19			
**Figure 2E_U373**					
Treatment (between columns)	3100099	3	1033366	F (3, 76) = 172.3	p<0.0001
Residual (within columns)	455870	76	5998		
Total	3555970	79			
**Figure 2E_LN229**					
Treatment (between columns)	742447	3	247482	F (3, 76) = 349.1	p<0.0001
Residual (within columns)	53885	76	709		
Total	796332	79			

DF: degrees of freedom; SS: sum of squares; MS: mean square (=SS/DF); F: F distribution value; *p*: probability.

Table S3. ANOVA analysis table of direct co-culture study data presented in Figure 3.

ANOVA table	SS	DF	MS	F (DFn, DFd)	*p* value
**Figure 3B_U373**					
Treatment (between columns)	9062	3	3021	F (3, 16) = 25.51	*p*<0.0001
Residual (within columns)	1895	16	118.4		
Total	10957	19			
**Figure 3B_LN229**					
Treatment (between columns)	4025	3	1342	F (3, 16) = 71.31	*p*<0.0001
Residual (within columns)	301.1	16	18.82		
Total	4326	19			
**Figure 3E_U373**					
Treatment (between columns)	4365	3	1455	F (3, 12) = 15.86	*p*=0.0002
Residual (within columns)	1101	12	91.73		
Total	5465	15			
**Figure 3E_LN229**					
Treatment (between columns)	1302	3	434	F (3, 12) = 25.27	*p*<0.0001
Residual (within columns)	206.1	12	17.18		
Total	1508	15			
**Figure 3F_U373**					
Treatment (between columns)	238348	3	79449	F (3, 76) = 81.61	*p*<0.0001
Residual (within columns)	73989	76	973.5		
Total	312337	79			
**Figure 3F_LN229**					
Treatment (between columns)	37601	3	12534	F (3, 76) = 44.27	*p*<0.0001
Residual (within columns)	21516	76	283.1		
Total	59116	79			

DF: degrees of freedom; SS: sum of squares; MS: mean square (=SS/DF); F: F distribution value; *p*: probability.

Table S4. ANOVA analysis table of *in vivo* study data presented in Figure 4.

ANOVA table	SS	DF	MS	F (DFn, DFd)	*p* value
**Figure 4C**					
Treatment (between columns)	35902	5	7180	F (5, 156) = 9.792	*p*<0.0001
Residual (within columns)	114391	156	733.3		
Total	150293	161			
**Figure 4D**					
Treatment (between columns)	35724	5	7145	F (5, 31) = 16.47	*p*<0.0001
Residual (within columns)	13447	31	433.8		
Total	49171	36			

DF: degrees of freedom; SS: sum of squares; MS: mean square (=SS/DF); F: F distribution value; *p*: probability.

Table S5. ANOVA analysis table of HUVEC data presented in Figure S4.

ANOVA table	SS	DF	MS	F (DFn, DFd)	*p* value
**Figure S4A_U373**					
Treatment (between columns)	2129	3	709.8	F (3, 12) = 16.39	*p*=0.0002
Residual (within columns)	519.8	12	43.31		
Total	2649	15			
**Figure S4A_LN229**					
Treatment (between columns)	3142	3	1047	F (3, 12) = 37.74	*p*<0.0001
Residual (within columns)	333	12	27.75		
Total	3475	15			
**Figure S4B_U373**					
Treatment (between columns)	169416	3	56472	F (3, 36) = 45.07	*p*<0.0001
Residual (within columns)	45107	36	1253		
Total	214523	39			
**Figure S4B_LN229**					
Treatment (between columns)	11829	3	3943	F (3, 36) = 19.05	*p*<0.0001
Residual (within columns)	7451	36	207		
Total	19279	39			
**Figure S4C_U373**					
Treatment (between columns)	15993	3	5331	F (3, 16) = 8.949	*p*=0.0010
Residual (within columns)	9531	16	595.7		
Total	25525	19			
**Figure S4C_LN229**					
Treatment (between columns)	14253	3	4751	F (3, 16) = 23.51	*p*<0.0001
Residual (within columns)	3233	16	202.1		
Total	17486	19			

DF: degrees of freedom; SS: sum of squares; MS: mean square (=SS/DF); F: F distribution value; *p*: probability.

Table S6. ANOVA analysis table of synergistic effects data presented in Figure S5.

ANOVA table	SS	DF	MS	F (DFn, DFd)	*p* value
**Figure S5A**					
Treatment (between columns)	4823	4	1206	F (4, 15) = 56.40	*p*<0.0001
Residual (within columns)	320.7	15	21.38		
Total	5143	19			
**Figure S5B**					
Treatment (between columns)	54033	4	13508	F (4, 15) = 44.83	*p*<0.0001
Residual (within columns)	4520	15	301.3		
Total	58553	19			
**Figure S5C**					
Treatment (between columns)	4652	4	1163	F (4, 15) = 40.53	*p*<0.0001
Residual (within columns)	430.4	15	28.7		
Total	5083	19			

DF: degrees of freedom; SS: sum of squares; MS: mean square (=SS/DF); F: F distribution value; *p*: probability.

Table S7. ANOVA analysis table of effect of Tiplaxtinin and Reparixin on EC cultured in evCM data presented in Figure S6.

ANOVA table	SS	DF	MS	F (DFn, DFd)	*p* value
**Figure S6A_left panel_U373**					
Treatment (between columns)	192.3	2	96.17	F (2, 9) = 3.973	*p*=0.0580
Residual (within columns)	217.8	9	24.2		
Total	410.2	11			
**Figure S6A_left panel_LN229**					
Treatment (between columns)	146.1	2	73.07	F (2, 9) = 3.862	*p*=0.0615
Residual (within columns)	170.3	9	18.92		
Total	316.4	11			
**Figure S6A_right panel_U373**					
Treatment (between columns)	52.53	2	26.27	F (2, 9) = 0.4928	*p*=0.6265
Residual (within columns)	479.7	9	53.3		
Total	532.2	11			
**Figure S6A_right panel_LN229**					
Treatment (between columns)	400.3	2	200.2	F (2, 9) = 2.927	*p*=0.1049
Residual (within columns)	615.5	9	68.38		
Total	1016	11			
**Figure S6B_U373**					
Treatment (between columns)	16.99	2	8.493	F (2, 9) = 0.06785	*p*=0.9349
Residual (within columns)	1127	9	125.2		
Total	1144	11			
**Figure S6B_LN229**					
Treatment (between columns)	61.04	2	30.52	F (2, 9) = 1.005	*p*=0.4037
Residual (within columns)	273.3	9	30.37		
Total	334.3	11			
**Figure S6C_U373**					
Treatment (between columns)	290.9	2	145.4	F (2, 27) = 0.1993	*p*=0.8205
Residual (within columns)	19704	27	729.8		
Total	19995	29			
**Figure S6C_LN229**					
Treatment (between columns)	160.1	2	80.03	F (2, 27) = 0.2650	*p*=0.7692
Residual (within columns)	8154	27	302		
Total	8314	29			
**Figure S6D_U373**					
Treatment (between columns)	396.2	2	198.1	F (2, 12) = 0.4023	*p*=0.6775
Residual (within columns)	5909	12	492.4		
Total	6305	14			
**Figure S6D_LN229**					
Treatment (between columns)	46.16	2	23.08	F (2, 12) = 0.05547	*p*=0.9463
Residual (within columns)	4993	12	416.1		
Total	5039	14			

DF: degrees of freedom; SS: sum of squares; MS: mean square (=SS/DF); F: F distribution value; *p*: probability.

A

B

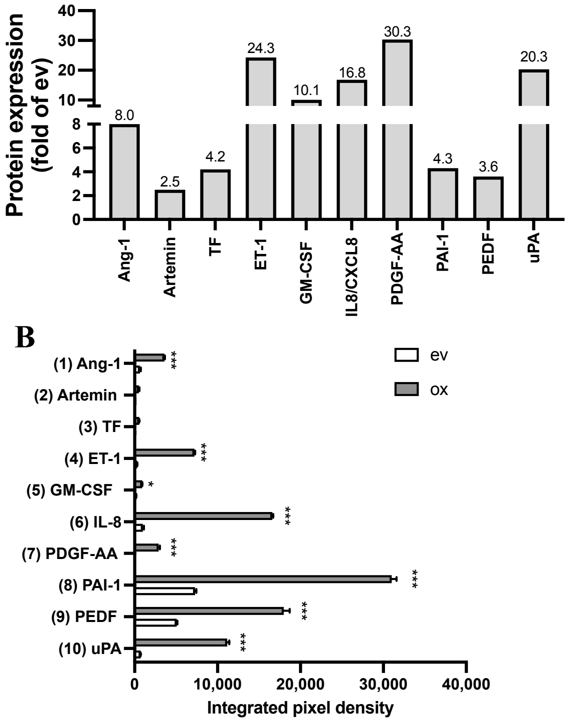

Figure S1. Semi-quantification of the blots of angiogenesis array. (A) The fold change of protein expression of 10 upregulated (≥ 2-fold) angiogenesis-related proteins. **(B)** The abundance of 10 upregulated proteins by integrated pixel density. PAI-1 and IL-8 showed the most abundant among all upregulated proteins. *, $p < 0.05$; ***, $p < 0.001$, compared with ev.

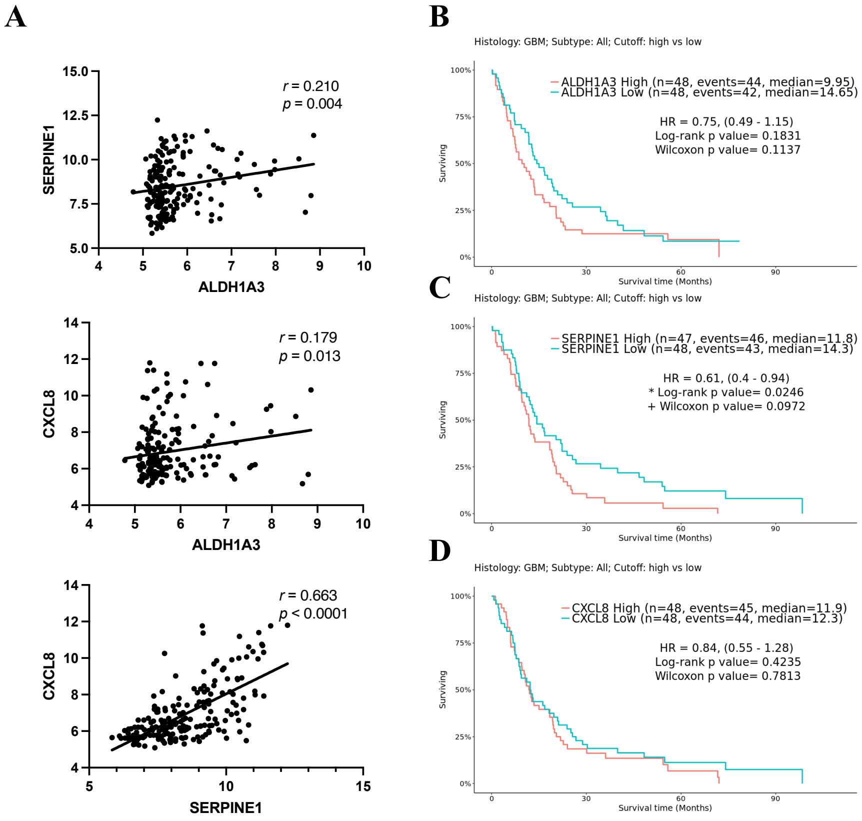

Figure S2. The expression of ALDH1A3, PAI-1 and IL-8 are associated with poor prognosis in GBM. (A) Correlation among ALDH1A3, SERPINE1 (PAI-1) and CXCL8 (IL-8) using the LeeY dataset from GlioVis. Pearson correlation was employed. **(B-D)** Kaplan-Meier survival curve of ALDH1A3 (**B**), SERPINE1 (**C**) and CXCL8 (**D**) with optimal cutoff for high vs low expression. A higher expression of ALDH1A3, SERPINE1 and CXCL8 were associated with a shorter OS time. All data were downloaded from GlioVis.

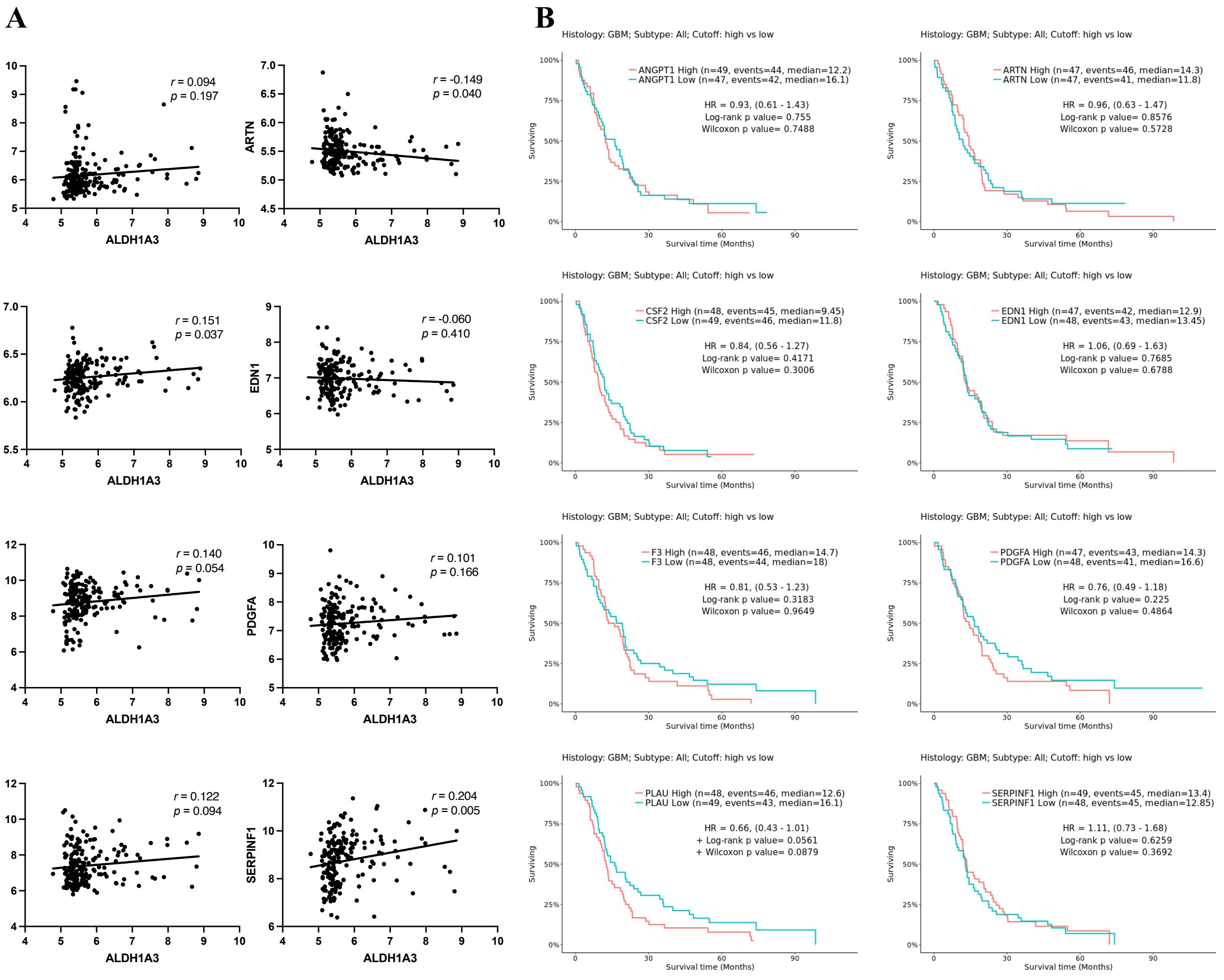

Figure S3. The expression and survival correlations of ALDH1A3 and angiogenesis factors in GBM. (A) Expression correlation between ALDH1A3 and ANGPT1 (Ang-1), ARTN (artemin), CSF2 (GM-CSF), EDN1 (ET-1), F3 (TF), PDGFA (PDGFAA), PLAU (uPA) and SERPINF1 (PEDF) in the LeeY dataset from GlioVis. Pearson correlation was employed. **(B)** Kaplan-Meier survival curve of each gene in (**A**) with optimal cutoff for high vs low expression. All data were downloaded from GlioVis.

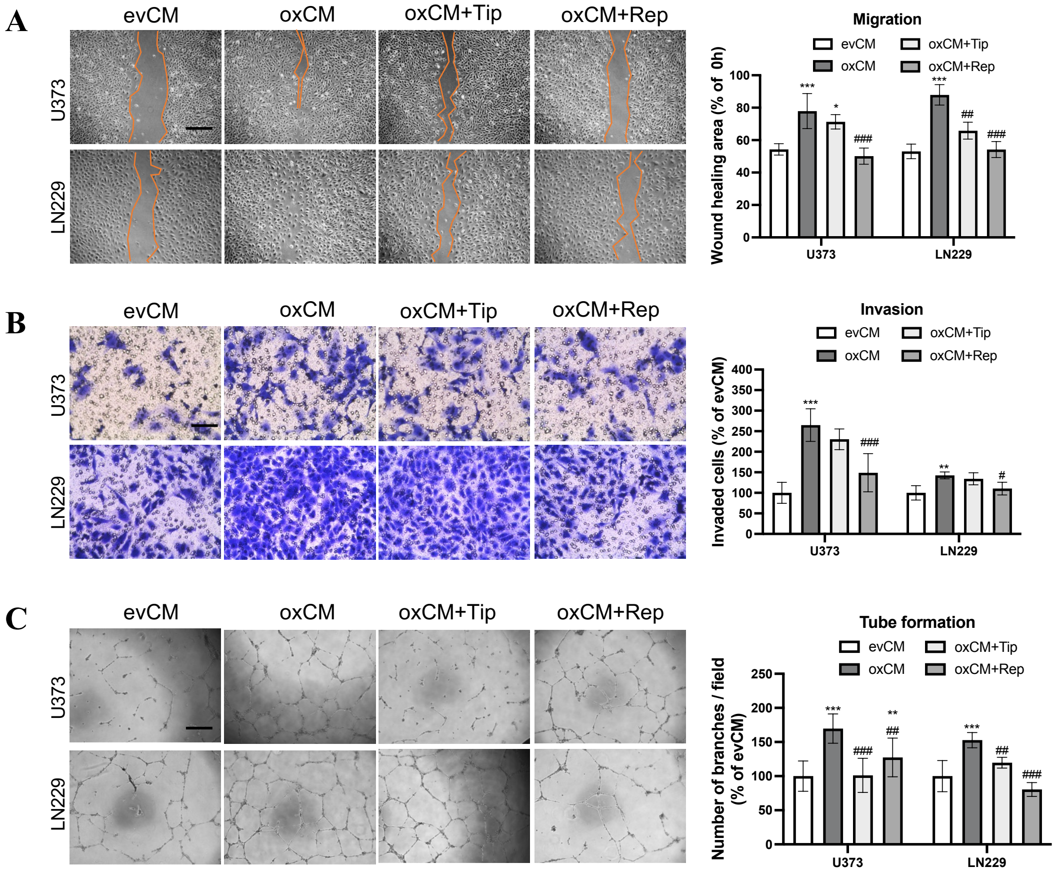

Figure S4. Indirect co-culture study of oxGBMs with HUVECs. Indirect co-culture was performed by culture of HUVEC in a conditioned medium (CM) containing the media derived from evGBM or oxGBM cells and ECGM in a ratio of 1:1. Tiplaxtinin (Tip, 30 μM) and Reparixin (Rep, 1 μM) or vehicle DMSO (0.1%) was added to CM followed by EC behavior study. All data were reproduced in three independent experiments. **(A) Scratch assay in HUVECs.** Left panel: images were acquired 24 h after scratching. Scale bar: 200 μm. Right panel: quantitative analysis. Culture of HUVEC with oxCM significantly promoted HUVEC migration, which was reversed by the treatment of Tiplaxtinin and Reparixin, respectively. **(B) Transwell invasion assay in HUVECs.** Left panel: Representative images of invaded cells were acquired after 24 h of incubation. Scale bar: 100 μm. Right panel: quantitative analysis. Culture of HUVEC with oxCM accelerated HUVEC invasion. This effect was significantly inhibited by the treatment of Reparixin but not by Tiplaxtinin. **(C) Tube formation assay in HUVECs.** Left panel: representative images of tube formation. Scale bar: 200 μm. Right panel: quantitative analysis of branching points per field. Tube formation in HUVEC was stimulated by the incubation with oxCM, which was completely diminished by both inhibitors. *, $p < 0.05$; **, $p < 0.01$ and ***, $p < 0.001$, compared with evCM. #, $p < 0.05$; ##, $p < 0.01$ and ###, $p < 0.001$, compared with oxCM.

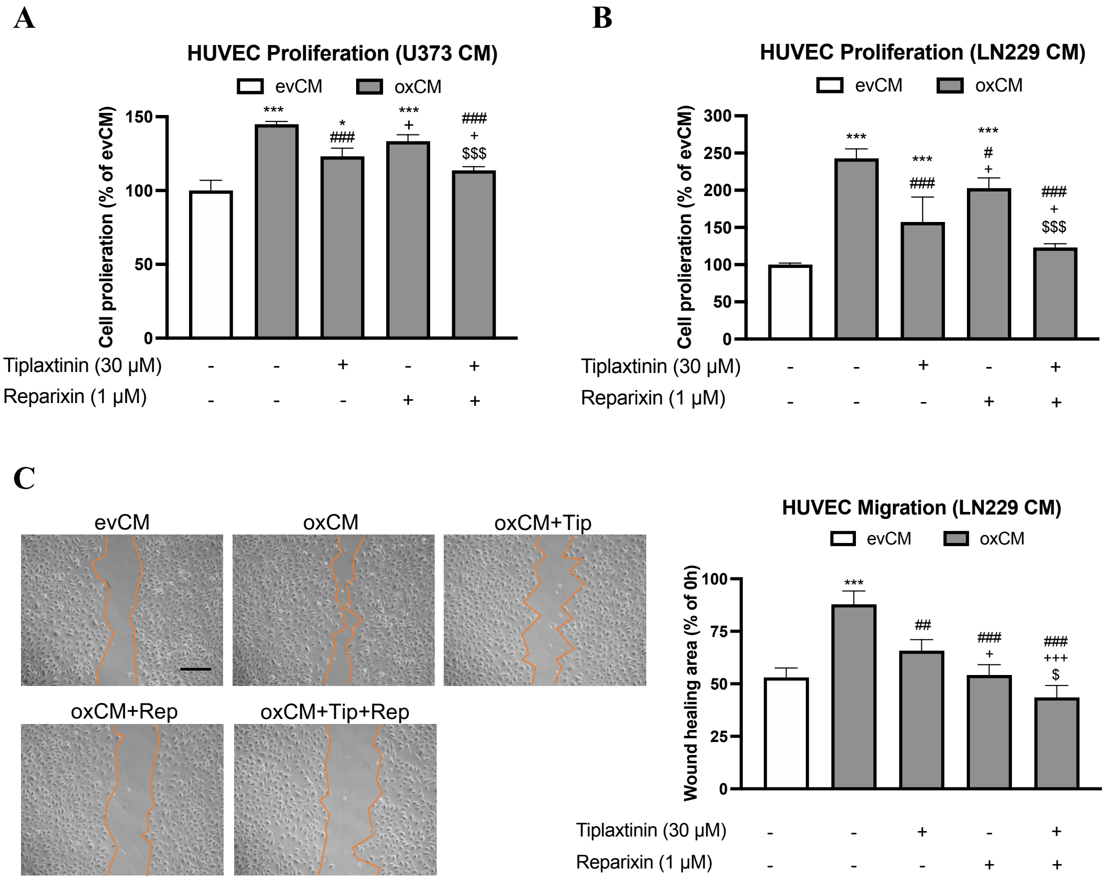

Figure S5. Synergistic effects of Tiplaxtinin and Reparixin on proliferation and migration of HUVECs in indirect co-culture model. Indirect co-culture was performed by culture of HUVEC in a conditioned medium (CM) containing the media derived from evGBM or oxGBM cells and ECGM in a ratio of 1:1. Tiplaxtinin (Tip, 30 µM) and Reparixin (Rep, 1 µM) or vehicle DMSO (0.1%) was added to CM followed by EC behavior study. **(A, B) Proliferation assay of HUVEC.** The combination of both inhibitors led to a stronger inhibition of proliferation in HUVECs treated with oxCM of U373 (A) and LN229 (B) respectively. **(C) Scratch assay in HUVECs.** Left panel: images were acquired 24 h after scratching. Scale bar: 200 µm. Right panel: quantitative analysis. The concurrent use of both inhibitors resulted in synergistic inhibition of migration in HUVECs treated with oxCM of LN229. *, $p < 0.05$ and ***, $p < 0.001$, compared with evCM. #, $p < 0.05$; ##, $p < 0.01$ and ###, $p < 0.001$, compared with oxCM. +, $p < 0.05$ and +++, $p < 0.001$ compared with oxCM+Tip. $, $p < 0.05$ and $$$, $p < 0.001$, compared with oxCM+Rep.

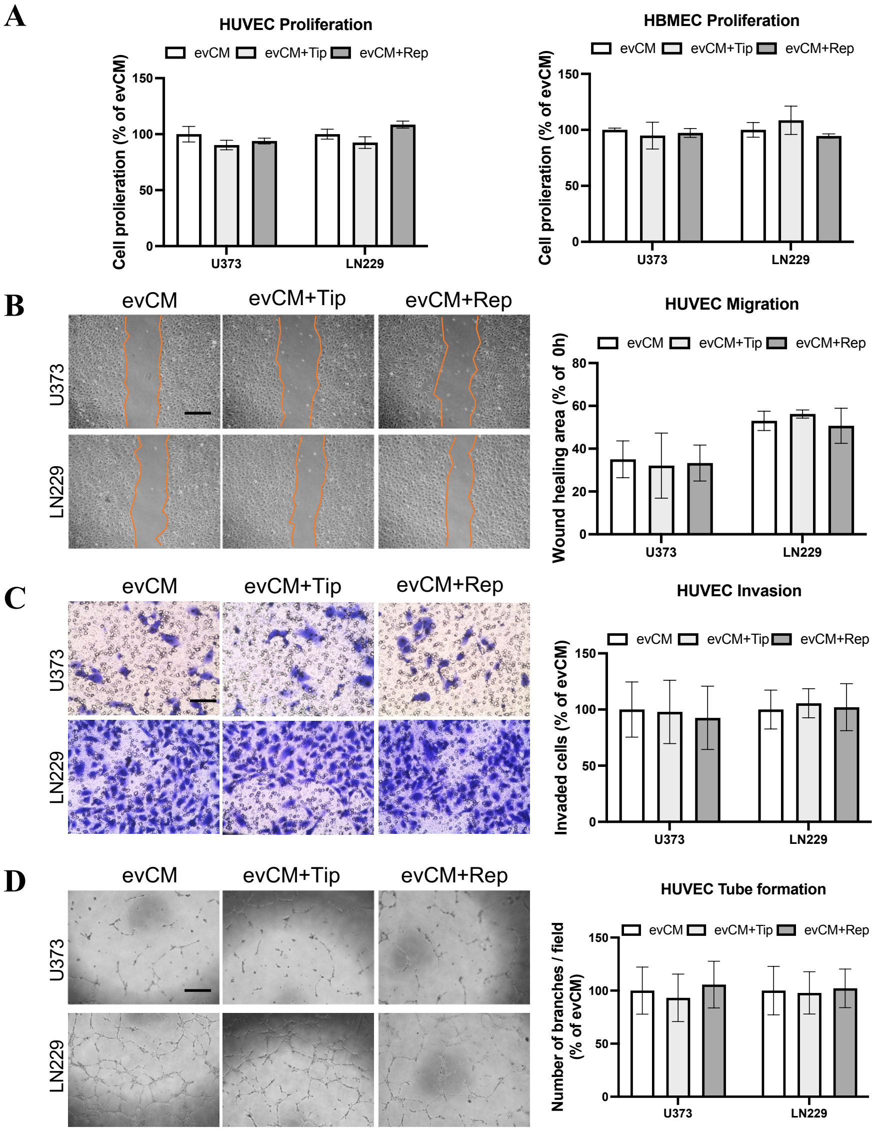

Figure S6. Effect of Tiplaxtinin and Reparixin on EC cultured in evCM. Indirect co-culture was performed by culture of ECs in a conditioned medium (CM) containing the media derived from evGBM cells and ECGM in a ratio of 1:1. Tiplaxtinin (Tip, 30 μM) and Reparixin (Rep, 1 μM) or vehicle DMSO (0.1%) was added to CM followed by EC behavior study. All data were reproduced in three independent experiments. **(A) Proliferation assay in HBMEC and HUVEC**. Tiplaxtinin and Reparixin did not alter the proliferation of evCM treated ECs. **(B) Scratch assay in HUVECs.** Left panel: images were acquired 24 h after scratching. Scale bar: 200 μm. Right panel: quantitative analysis. The migration of HUVECs treated with evCM remained unaffected by Tiplaxtinin and Reparixin. **(C) Transwell invasion assay in HUVECs.** Left panel: Representative images of invaded cells were acquired after 24 h of incubation. Scale bar: 100 μm. Right panel: quantitative analysis. Tiplaxtinin and Reparixin had no effect on the invasion of evCM treated ECs. **(D) Tube formation assay in HUVECs**. Left panel: representative images of tube formation. Scale bar: 200 μm. Right panel: quantitative analysis of branching points per field.

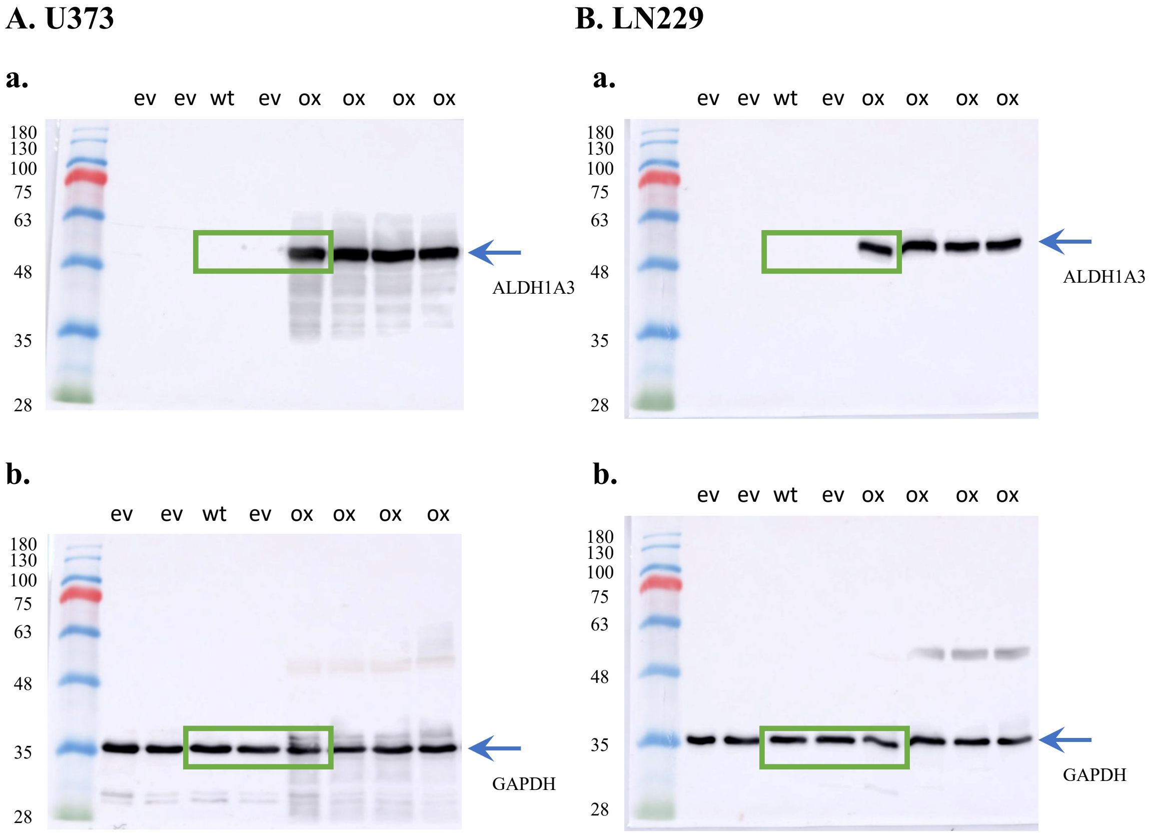

Figure S7. Original immunoblots for Figure 1B. U373 **(A)** and LN229 **(B)**. The blots shown in Figure 1B for ALDH1A3 (a) and GAPDH (b) were marked in boxes. Others were from different passages of cells, whose data were not included in Figure 1B.

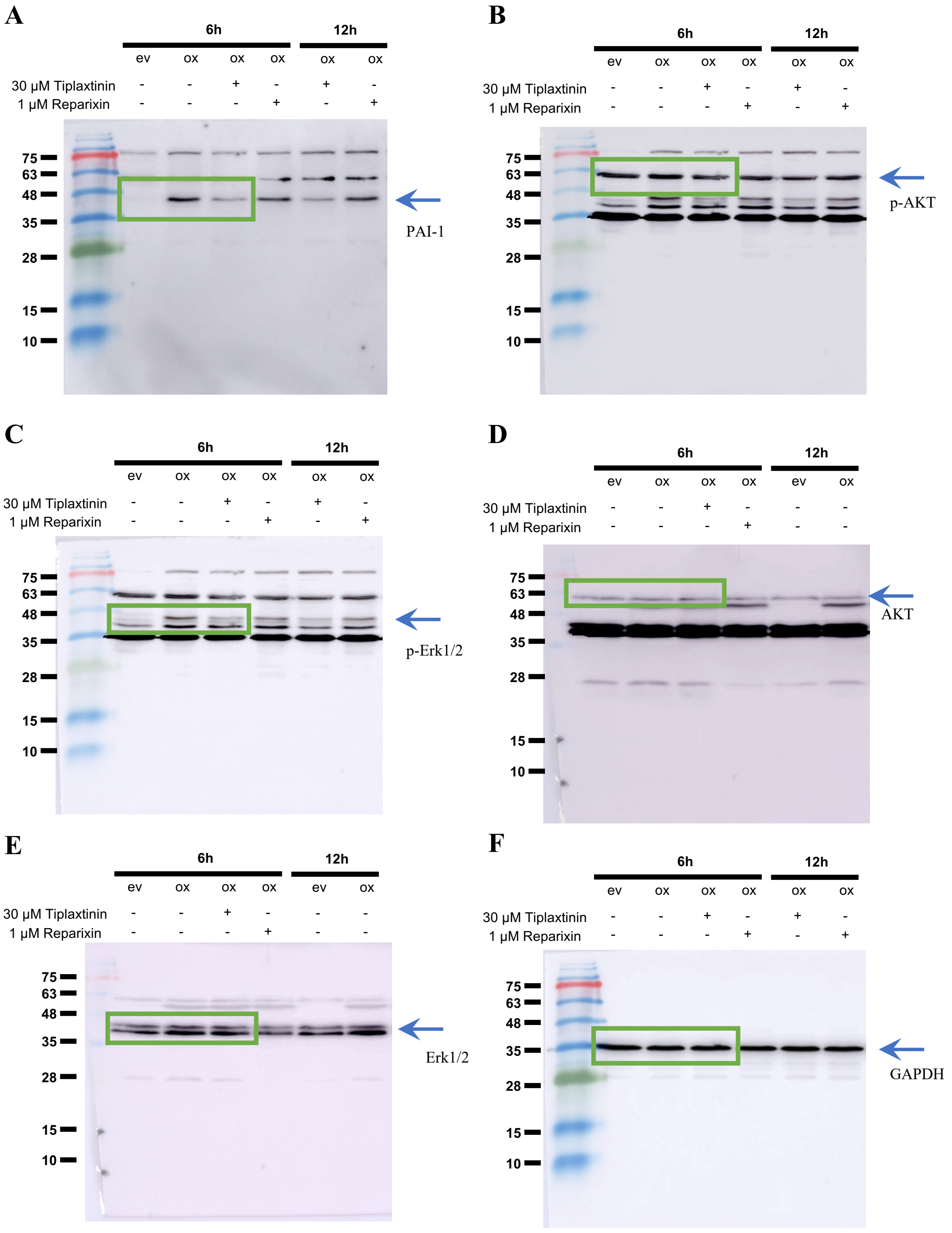

Figure S8. Original immunoblots for Figure 1F. U373 cells were collected after 6 h treatment of Tiplaxtinin (30 μM) for western blot. Another subset of cells was from different time points and the treatment of Reparixin (1 μM), whose data were not included in Figure 1F. The blots shown in Figure 1F for PAI-1 **(A)**, p-AKT **(B)**, p-Erk1/2 **(C)**, AKT **(D)**, Erk1/2 **(E)**, GAPDH **(F)** were marked in boxes.
